# Identification, function validation and haplotype analysis of salt-tolerant genes of lectin receptor kinase gene family in sorghum (*Sorghum bicolor* L.)

**DOI:** 10.3389/fgene.2024.1464537

**Published:** 2024-10-15

**Authors:** Li Mao, He Huazhuan, Gao Haiyan, Huang Wangqi, Cai Qizhe, Yan Guiyun, Cheng Qingjun, Liang Yinpei, Chen Xiuhua

**Affiliations:** ^1^ Shanxi Key Laboratory of Minor Crops Germplasm Innovation and Molecular Breeding, Shanxi Agricultural University, Taigu, China; ^2^ College of Life Sciences, Shanxi Agricultural University, Taigu, China; ^3^ College of Agriculture, Shanxi Agricultural University, Taigu, China; ^4^ Institute of Flower, Yunnan Academy of Agricultural Sciences, Kunming, China; ^5^ International Agriculture Research Institute, Yunnan Academy of Agricultural Sciences, Kunming, China

**Keywords:** L-type lectin receptor kinases, Sorghum bicolor, salt stress, function validation, haplotype

## Abstract

**Introduction:**

Salt stress is one of the significant challenges in sorghum production, greatly impacting the yield of *S. bicolor*. L-type lectin receptor kinases (*LLRKs*) are essential for plant stress tolerance, yet a comprehensive genome-level analysis in this species has not been conducted.

**Materials and methods:**

Members of the *SbLLRLK* gene family were identified using bioinformatics methods. Gene structures, motifs, and phylogenetic relationships were analyzed. Subsequently, expression profiles under various stress conditions were examined using transcriptome data. Furthermore, functional validation was performed through homologous gene alignment and transgenic analysis, focusing on the gene SORBI_3004G304700.

**Results:**

This study identified 49 *SbLLRLK* genes in *Sorghum bicolor*, categorized into four groups based on their lectin domain characteristics. Expression analyses revealed diverse patterns under salt, drought, and heat stresses. SORBI_3004G304700 was identified as a negative regulator of salt stress tolerance, with three unique haplotypes identified through haplotype analysis, suggesting a role in salt stress adaptation. Haplotype analysis of SORBI_3004G304700 revealed three unique haplotypes, with haplotype1 being the most prevalent, possibly due to selective advantages for salt stress tolerance.

**Discussion:**

These findings offer valuable insights into the biological research on the role of the LLRLK gene family in Sorghum bicolor’s response to salt stress. The functional characterization of SORBI_3004G304700 and the identification of haplotypes associated with salt tolerance provide valuable insights for molecular breeding and genetic enhancement of sorghum.

## 1 Introduction

Sorghum (*Sorghum bicolor* (L.) Moench), the world’s fifth-largest cereal crop, serves as a vital staple food for over 500 million individuals, primarily in arid and semi-arid regions (Ananda et al., 2020; Rashwan et al., 2021). However, the soil salinization resulting from human factors such as industrial pollution and unreasonable irrigation caused negative impact on sorghum yield and food production (Mukhopadhyay et al., 2021). Salinity-induced reductions in germination rates, seedling emergence, and flowering profoundly affected the overall yield potential of sorghum (Arif et al., 2020). Salinity stress disrupts cellular homeostasis, impairs nutrient uptake, and induces oxidative stress, all of which negatively impact sorghum growth and development (Mansour et al., 2021). Therefore, identification of key genes involved in salt stress tolerance equips researchers with molecular tools to alleviate this abiotic stress on sorghum production (Baye et al., 2022). Moreover, understanding the genetic basis allows for the targeted development of sorghum varieties with enhanced salt stress tolerance (Hao et al., 2021). This research contributes not only to sorghum production but also aligns with broader global efforts toward achieving food security in a changing climate (Amombo et al., 2022).

Plants respond to environmental changes through a variety of complex signaling systems involving receptor-like kinases (RLKs) (Zhu, 2002; Osakabe et al., 2013). Since the first RLK was discovered and characterized in maize in 1990 (Walker and Zhang, 1990), RLK has been identified in many plants (Shiu and Bleecker, 2001). Receptor-like kinases (RLKs) are categorized into 15 classes based on their extracellular domains (De Smet et al., 2009). Among these classes, lectin receptor-like kinases (*LRLKs*) are distinguished by their carbohydrate-binding lectin domains. *LRLKs* are integral components in a range of biological mechanisms, such as plant growth and development, immunity against diseases, self-incompatibility responses, and reactions to environmental stresses (Vaid et al., 2012; Sun et al., 2020). *LRLKs* have also been shown to be responsible for signal transduction from the outside to inside cells, and they are identified by their carbohydrate-binding N-terminal lectin domains and intracellular C-terminal kinase domain (Hervé et al., 1999; Lannoo and Van Damme, 2014). *LRLKs* exhibit significant variability in their extracellular domains, enabling them to perceive a diverse range of signals, including those associated with hormones and stress (Bouwmeester et al., 2011). *LRLKs* can be classified into three subgroups based on their extracellular lectin domains: C-type, G-type and L-type (*LLRLKs*) (Lagarda-Diaz et al., 2017; Passricha et al., 2019).

There is a complex network consisting of various pathways to regulate the response of plants to salt stress (Arif et al., 2020). Among these, the osmotic adjustment pathway, the SOS pathway, the reactive oxygen species (ROS) scavenging pathway, and the signal transduction pathway stand out as pivotal regulators in enhancing salt stress tolerance (Yang and Guo, 2018). Through the precise regulation of these pathways, plants are able to effectively address osmotic imbalances, reduce oxidative stress, and preserve cellular homeostasis, thus ensuring their survival and productivity in saline environments (Bano et al., 2022). Notably, the *LLRLKs* play a pivotal role in the signal transduction pathway, facilitating essential signaling processes that enhance the plant’s adaptive capacity to saline conditions (Sun et al., 2020). Many recent studies have demonstrated the significant role of *LLRLKs* in plants when resisting salt stress (Li et al., 2014; Sun et al., 2020). For example, the *LLRLK* genes are important in increasing the ability of soybean plants to resist salt stress by controlling the expression of genes that respond to salt (Zhang et al., 2022). Many other *LLRLKs* were considered as factors in the mediation of abiotic stress resistance in *Arabidopsis* (Vaid et al., 2013). Likewise, *OsSIK1* (Os06g03970), a rice *LLRLK* protein, exhibited salt-inducible expression and functioned as a facilitator of salt stress tolerance (Ouyang et al., 2010). The introduction of a *LLRLK* (Os02g42780) resulted in a negatively regulated salt stress tolerance in rice, and the RNAi plants showed an increased tolerance to salinity (Li et al., 2014). It has been demonstrated that *OsLLRLK* is responsible for salt stress tolerance in rice through the reprogramming of stress-responsive metabolic pathways (Passricha et al., 2020). With the accumulation of sequencing technology and genome transcriptome data, comprehensive investigations of the *LLRLK* gene family have been carried out in various species (Ouyang et al., 2010). Such analyses have encompassed species such as rice (Passricha et al., 2020), *Arabidopsis* (Shiu and Bleecker, 2003), potato (Zhang et al., 2020), etc. (Lv et al., 2020). However, research on the genome-wide identification and evolutionary dynamics of the *LLRLK* gene family in sorghum is limited and scarce. Considering the significant role of the *LLRLK* gene family in plant salt stress tolerance, comprehensive studies of this gene family in sorghum will be of great significance.

The objectives of this research are to: (a) precisely identify and characterize *LLRLK* genes in Sorghum bicolor; (b) predict the potential functions of these *LLRLK* genes through prior studies, homology analysis, and expression pattern assessments; (c) investigate candidate genes linked to salt tolerance via homologous comparisons and transgenic analyses; and (d) conduct haplotype analysis on salt-tolerance genes to identify their advantageous alleles. These results have the potential to enhance comprehension of the regulatory mechanisms that govern the reaction of the *LLRLK* gene family to environmental stressors, shedding light on the biological functions of *LLRLK* genes in *S. bicolor* under salt stress, and it can provide a valuable reference for future biological investigations on this species.

## 2 Materials and methods

### 2.1 Identification of *LLRLK* family members in *Sorghum bicolor*

The annotated protein sequences of *S. bicolor* (v3.51) was obtained from Ensemble plants database (https://plants.ensembl.org/index.html). The Lectin_legB domains (PF00139) and Pkc_like domains (PF06176) were acquired as profile HMMs from the Pfam database (http://pfam-legacy.xfam.org/). These HMMs were utilized to identify members of the *LLRLK* family within the annotated *Sorghum bicolor* protein sequences (https://ftp.ebi.ac.uk/ensemblgenomes/pub/release-59/plants/fasta/sorghum_bicolor/pep/). The search was performed using the HMMER software package (http://hmmer.org/) (Finn et al., 2011) with default parameters. Additionally, the *Arabidopsis LLRLKs* were selected as queries to conduct a blast search against the *Sorghum bicolor* protein sequences, using an E-value threshold of ≤1e-5. After validation with the NCBI-CDD (https://www.ncbi.nlm.nih.gov/Structure/cdd) (Marchler-Bauer et al., 2017), sequences presumed to be *SbLLRLK* but lacking the full *LLRLK* motif (Lectin_legB_N and Pkc_like_C) were excluded. Additionally, redundant entries were also removed by performing multiple sequence alignments with Clustal X (http://www.clustal.org/clustal2/) (Chenna et al., 2003) to refine the dataset for further analysis.

### 2.2 Physicochemical characteristics, chromosomal distributions, and gene duplication events among the members of the *SbLLRLK* gene family

The *SbLLRLK* gene structures, formatted in GFF3, were obtained from the Ensemble Plants database. Visualization of these structures was accomplished with the CFVisual v2.1 tool (https://github.com/ChenHuilong1223/CFVisual/releases/tag/CFVisual). For the identification of motifs within the *SbLLRLK* proteins, the MEME suite (https://meme-suite.org/meme/) was employed. The motif width was set to range from 6 to 200 residues, allowing for any number of repetitions, and setting the maximum number of motifs to detect at 20. The Gene Structure Display Server 2.0 (https://gsds.gao-lab.org/Gsds_help.php) (Hu et al., 2015) was then engaged to graphically represent the conserved domains of these proteins. The predictive modeling of the *SbLLRLK* protein’s three-dimensional structure was carried out using the AlphaFold platform (https://alphafold.com/) (Huang et al., 2024).

### 2.3 Phylogenetic analysis and classification of *SbLLRLK* family members

The reference protein sequences for the *LLRLK* gene family from *Arabidopsis thaliana*, rice (*Oryza sativa* L.), wheat (*Triticum aestivum* L.) and barley (*Hordeum vulgare* L.) were retrieved according to the previous studies (Vaid et al., 2012; Shumayla et al., 2016; Ahmed et al., 2024). Finally, the total 49 *SbLLRLK*, 38 *AtLLRLK*, 72 *OstLLRLK*, 84 *TaLLRLK* and 49 *HvLLRLK* protein sequences (the longest transcripts were employed) were obtained to establish a phylogenetic tree across species. The sequences were aligned using MUSCLE 3.8 (https://github.com/rcedgar/muscle) (Yang et al., 2024) with default settings. The phylogenetic analysis was carried out employing the neighbor-joining method in MEGA11 (https://www.megasoftware.net/) (Hall, 2013), validated with 1,000 bootstrap replicates. Consequently, the *LLRLKs* were confirmed into sub-types and the phylogenetic tree was subsequently redrawn and enhanced by ggtree package in the R program (Yu et al., 2017). Moreover, the phylogenetic tree of the *LLRLK* proteins from sorghum was also constructed and analyzed. To accomplish this, a multiple sequence alignment of the 49 *SbLLRLK* proteins was performed, followed by the construction of the phylogenetic tree using MUSCLE 3.8 (https://github.com/rcedgar/muscle) (Yang et al., 2024).

### 2.4 Synteny analysis of the *SbLLRLK* genes and other plant species’ proteins

The protein sequences of *Sorghum bicolor*, *A. thaliana*, and rice (*O. sativa* L.) were acquired from Ensemble Plants databases. The makeblastdb program (https://rdrr.io/github/mhahsler/rBLAST/man/makeblastdb.html) was then employed to create local databases for each species. Subsequently, all the *SbLLRLK* protein sequences were subjected to a pairwise comparison against the sequences of two other species, utilizing the BLASTp algorithm (https://ftp.ncbi.nlm.nih.gov/blast/executables/blast+). The analysis of syntenic relationships was conducted with the MCScanX tool (https://github.com/wyp1125/MCScanX) (Wang et al., 2012).

### 2.5 Expression profile analysis

The sorghum transcriptome data under different abiotic stresses treatment were retrieved from the MOROKOSHI Sorghum database (http://sorghum.riken.jp/Home.html) (Makita et al., 2015). The plants were subjected to different treatment such as salinity (300 mM NaCl), drought (260 μM mannitol), heat (35°C), PEG, and ABA (50 μM). Heatmap visualizations of the gene Log2 (FPKM +1) values were generated using the complex pheatmap package in the R program (Gu, 2022).

In a salinity stress study, sorghum seedlings at the age of 10 days were subjected to a 200 mM NaCl solution for intervals of 0, 3, 6, and 9 h. Then the seedlings were rapidly frozen in liquid nitrogen and preserved at −80°C for further analysis. The leaves of the plants were quickly frozen in liquid nitrogen and placed in a −70°C freezer for RNA extraction. RNA was extracted using a commercial kit from Tiangen (China, catalog number DP412), with the RNA then being concentrated and purified in preparation for cDNA synthesis. This was achieved using a reverse transcription kit (Tiangen, China, catalog number FP205-01), following the manufacturer’s guidelines. The cDNA samples were diluted five times and used as the templates for qPCR. Quantitative real-time PCR (qPCR) was executed with a specific mix (Tiangen, China; code no. FP205-01) on an ABI 700 system (Applied Biosystems, United States), following the protocol provided by the kit’s manufacturer. The reaction conditions comprised 95°C for 2 min, followed by 40 cycles of 95°C for 30 s, 60°C for 35 s, and 72°C for 30 s. The gene expression levels were analyzed using the 2^−ΔΔCT^ method. The gene expression levels of SORBI_3004G304700 were standardized against the *SbCYP* (Xm_002453800) gene (Sudhakar Reddy et al., 2016). Primer details are outlined in the Supplementary Table S1, datasheet 3.

### 2.6 Subcellular localization of *SbLLRLKs*

To conduct the subcellular localization analysis of the *SbLLRLK* protein in transgenic tobacco, the SORBI_3004G304700 gene was chosen as a representative. The cDNA fragment of SORBI_3004G304700 without stop codon was amplified using the GFPF and GFPR primers (Supplementary Table S1, datasheet 3).The PCR fragment was cloned into the PCEGFP vector, which was digested by Spe1 to generate the expression cassette with an In-Fusion Clone Kit (Vazyme Nanjing, China). The coding sequence of SORBI_3004G304700 was fused in frame to the 5′ends of the green fluorescent protein (GFP) (Supplementary Figures S4, S5). After the nucleotide sequence of the constructed vector was confirmed by PCR and DNA sequencing, 35S:SORBI_3004G304700: GFP was introduced to the epidermal cells of tobacco (*Nicotiana benthamiana*) via the Agrobacterium-mediated method (More et al., 2019). The 35S: GFP was used as the control. After the Agrobacterium and tobacco leaves were co-cultured for 72 h, a subcellular localization of the target gene was conducted by observing the expression of green fluorescent proteins (GFPs) in the tobacco leaf cells. To explore the cellular distribution of the SORBI_3004G304700: GFP fusion proteins in transgenic tobacco plants, leaf specimens were mounted on slides for microscopic analysis. The examination was conducted using a high-resolution microscope (Olympus, Tokyo, Japan). Images were acquired using a ×20 objective lens with 488 nm excitation and 509 nm emission filters, and the scan speed was 7 lines per second. The images obtained were then transferred to dedicated imaging software (FV10-ASW 1.7A, Olympus, Tokyo, Japan) for detailed analysis and assessment.

### 2.7 Function validation of SORBI_3004G304700 by tobacco transformation

The tobacco transformation was conducted based on a recently published protocol, with a few modifications (Nguyen et al., 2015). Healthy and young leaves of tobacco seedlings were used as transgenic explants, and they were cut into 1–2 cm squares. The explants were then placed into a Murashige and Skoog (MS) medium, and then incubated in a plant growth chamber at 25°C for 3 days. After that, the explants were incubated with 2 mL of Agrobacterium GV3101, which had been transformed by PCEGFP: SORBI_3004G304700 for 8 minutes. The explants were washed thrice with distilled water and then transferred to a MS medium containing 50 mg/L of hygromycin (Supplementary Figure S6A). Four weeks later, the screened explants were transferred to shoot regeneration MS medium containing 1.5 mg/L 6-BA (6-Benzylaminopurine) and 200 mg/L Carbethycin (Supplementary Figure S6B). Four weeks later, the regenerated plants were moved to a MS medium containing 0.5 mg/L of 1-naphthalene acetic acid (NAA) after the roots were induced. In addition, after the roots were induced, the plants were also transplanted into soil and transferred to a greenhouse with a 16 h light and 8 h dark photoperiod at 25°C ± 2°C (Supplementary Figure S6C). The T2 generation of tobacco plants was used for subsequent experiments. The putative transgenic plants (TP1 and TP2) were confirmed by PCR and QRT-PCR (Supplementary Figure S7). The *Nbactin* was used for reference gene. The PCR and QRT-PCR primers used in the experiment are listed in Supplementary Table S1, datasheet 3. The negative control for the PCR and and QRT-PCR reaction was the wild-type (WT) gene (Supplementary Figure S7). Each sample had three biological replications.

After the transgenic plants were generated, the wild-type (WT) and T2 generation of transgenic seeds were surface-sterilized with 70% (v/v) ethanol, followed by 8% NaClO (v/v), and were sown on filter paper that was supplemented with a Hoagland solution containing 0 and 200 mM of NaCl. The plates were placed under greenhouse conditions, and the H_2_O_2_ and superoxide accumulation were measured after 10 days. The 3, 3′-diamin-obenzidine (DAB) and nitrobluetetrazolium (NBT) staining was carried out as described earlier (Daudi and O’Brien, 2012) to determine the H_2_O_2_ and superoxide accumulation in salinity-stressed plants. The seedlings of the 10-day-old wild-type plants (WT) and transgenic plants (TP1 and TP2) under normal and salt-stress conditions (200 mM of NaCl) were stained under dark conditions for 24 h until dark spots could be observed. Then, the leaves were boiled in 95% ethanol, kept in 70% ethanol at room temperature, and were then photographed. The accumulation of H_2_O_2_ and superoxide were evaluated by the stain intensity of DAB and NBT, which were determined and calculated by ImageJ software (https://imagej.net/ij/). Each sample had three biological replications. Haplotype analysis of SORBI_3004G304700 associated with salt stress tolerance.

### 2.8 Haplotype analysis of SORBI_3004G304700 associated with salt stress tolerance

The 31 sorghum cultivars (Supplementary Table S1, datasheet 5) used for the assessment of salt stress tolerance were collected globally by our research team. Evaluation of the salt stress tolerance of each sorghum variety was conducted by assessing the survival rate during the seedling stage in 2022 and 2023, as reported previously (Mansour et al., 2021). Single nucleotide polymorphisms (SNPs) employed for haplotype analysis were obtained from the SorghumBase database (https://sorghumbase.org/) (Tao et al., 2021). The identification of haplotypes and their correlation with salt stress tolerance phenotype were analyzed by the geneHapR tool (Zhang et al., 2023). The following parameters were applied: heterozygous sites and samples with missing genotypes were filtered out. The significance analysis method used was Duncan’s test, with a minimum sample size of 5 for significance calculations. The maximum size of indels displayed was 5 base pairs for both deletions and insertions.

## 3 Results

### 3.1 Identification of *SbLLRLKs* in sorghum

**FIGURE 1:**
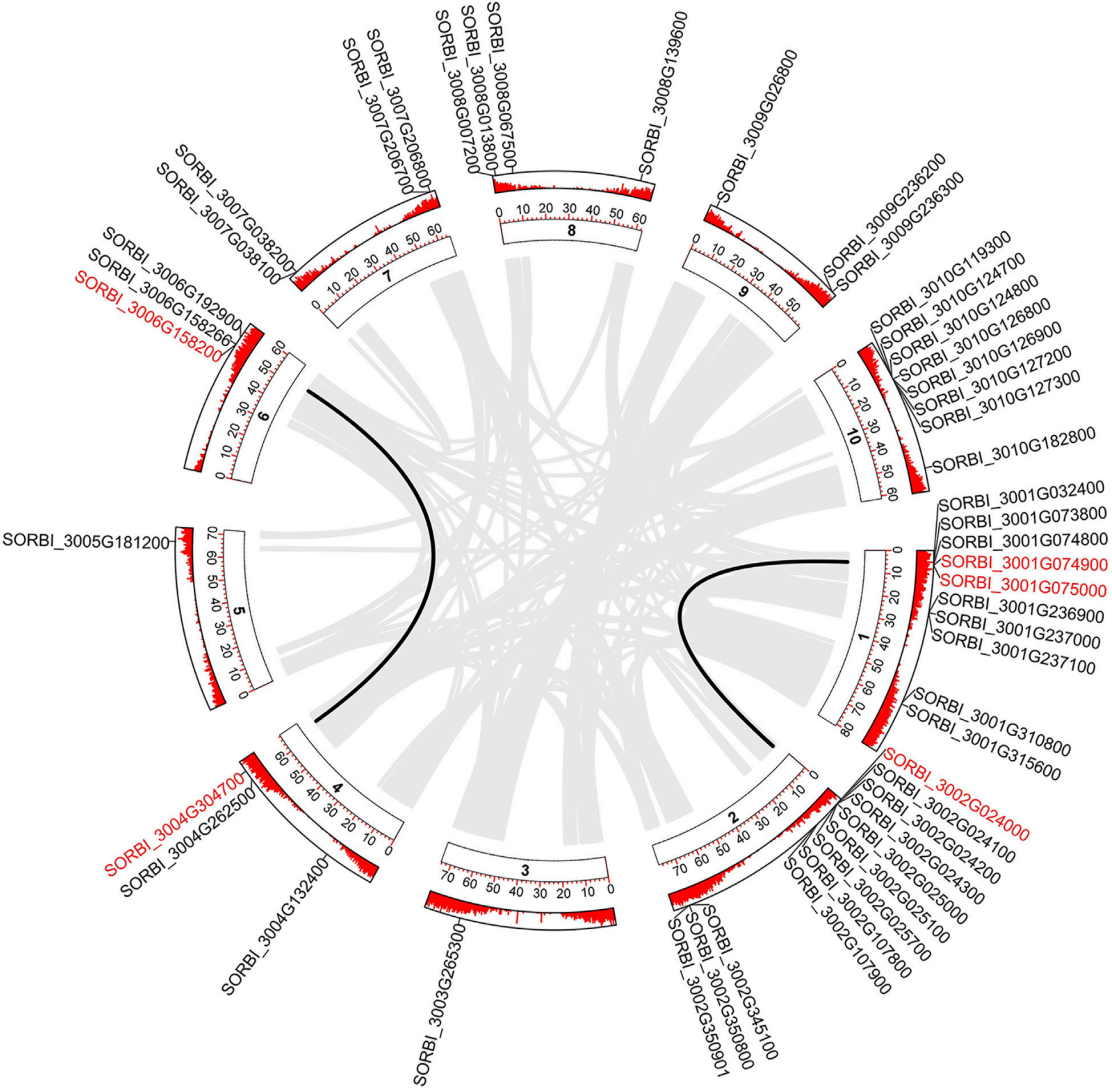
Segmental duplication of the *SbLLRLK* genes in sorghum. The gray lines in the background represent the segmental duplication in the whole genome. Black lines represent the *SbLLRLK* gene pairs with segmental duplication. The inner circle containing numbers represents the different chromosomes of sorghum, while the outer circle represents the gene density on the different chromosomes, where each short red line represents a gene locus.

In total, 49 members of *SbLLRLKs* were identified in the sorghum genome (Figure 1). The coding sequence (CDS) lengths for the candidate *SbLLRLKs* ranged from 1,440 bp (SORBI_3002G024300) to 2,379 bp (SORBI_3007G206800), encoding 480 amino acids (SORBI_3002G024300) to 793 amino acids (SORBI_3007G206800), respectively. These proteins had molecular weights ranging from 53,466.47 to 86,464.1 Da (Supplementary Table S1, datasheet 1). The calculated isoelectric points of the *SbLLRLK* gene products varied from 5.36 to 8.77, indicating their potential functional roles in diverse cellular microenvironments. Analysis of the grand average of hydropathicity (GRAVY) data revealed that 32 *SbLLRLK* genes are likely hydrophilic proteins (Supplementary Table S1, datasheet 1).

Among these *SbLLRLKs*, 49 genes were unevenly distributed in the 10 chromosomes of sorghum (Figure 1). The chromosome mapping analysis revealed that *SbLLRLKs* were distributed with notable clustering on chromosomes 1, 2, and 10 (Figure 1). However, chromosome 3 and 5 contained only one *SbLLRLK* gene (SORBI_3003G265300 and SORBI_3005G181200) (Figure 1). The subcellular localization prediction indicated that the majority of *SbLLRLK* genes were located in the cell membrane, while a few members were found in the chloroplast and nucleus. Additional information on the predicted physical and chemical properties of *SbLLRLKs* can be found in Supplementary Table S1, datasheet 1.

### 3.2 Structural analysis of the conserved domains in *SbLLRLK* genes

The sequences and positions of the kinase domains and lectin domains in the 49 *SbLLRLK* members were detected and annotated (Supplementary Figures S1, S2). The composition of the secondary structures was greatly different between the two domains. A total of 11 α-helices, 9 β-sheets, and 5 η-helices were annotated in the kinase domain (Supplementary Figure S2), and these facilitated the function of the kinase domain in the *SbLLRLK* proteins. In comparison, 18 β-sheets were found in the lectin domain, while only 2 α-coils and 1 η-helix were found in the same domain (Supplementary Figure S1).

The analysis of multiple sequence alignment for the two domains revealed that the conserved amino acids were discontinuous (Supplementary Figures S1 and S2). There were many striking features in the two domains and these features are relatively conserved within the family members. For example, the most obvious feature (DFGL) in the kinase domain was located between β8 and β9 (Supplementary Figure S1), while the GxGxxG between η1 and β2 in the kinase domain was also conspicuous (Supplementary Figure S2). In addition, the threonine (T) and phenylalanine (F) at the position of 361 and 364 were also found and were considered as the active position of *LLRLK* genes in other species such as *Arabidopsis* and rice (Vaid et al., 2013), thereby suggesting that these two sites are extremely conserved in plants (Supplementary Figure S2).Despite that the sequences of the lectin domain in *SbLLRLKs* were relatively variable, there is still a conserved glycine (G) at the position of 253 in the lectin domain (Supplementary Figure S1).These all suggested that *SbLLRLKs* were relatively structurally conserved during the evolution of sorghum.

### 3.3 Duplication analyses of the *SbLLRLK* genes in sorghum

The investigation of gene duplication events within the sorghum genome revealed distinct categories, which consisted of 6,837 single-copy genes, 11,766 dispersed genes, 21,585 tandem duplications, 4,866 WGDs or segmental duplications, as well as 2,056 adjacent but non-contiguous repetitive genes in the sorghum genome (Supplementary Table S1, datasheet 2).

Of all the 49 *SbLLRLK* genes, only 5 genes were found to be involved in duplication events, comprising of 3 pairs of segmental duplications and 1 pair of tandem duplication (Figure 1; Table 1). The Ka/Ks values of all the duplicated gene pairs were less than 1 (Table 1), indicating that these genes undergo different levels of purifying selection during the process of evolution. This fact also suggested that *SbLLRLKs* were relatively conserved during the evolution of sorghum. Furthermore, gene duplication resulted in evident 3D structural similarity between the duplicated gene and the original gene, such as SORBI_3006G158200 and SORBI_3004G304700, as well as SORBI_3001G074900 and SORBI_3002G024000 (Supplementary Figure S3).

**TABLE 1:** The Ka/Ks values of the *SbLLRLK* gene pairs in sorghum.

Seq_1	Seq_2	Ka	Ks	Ka/Ks	Duplicated type
SORBI_3006G158200	SORBI_3004G304700	0.21140927	0.697052665	0.303290241	tandem duplication
SORBI_3001G074900	SORBI_3002G024000	0.320601806	0.66864807	0.479477651	segmental duplication
SORBI_3001G075000	SORBI_3002G024000	0.271995114	0.568266931	0.478639701	segmental duplication
SORBI_3001G074900	SORBI_3001G075000	0.326909004	0.700278919	0.466826853	segmental duplication

Note: WGD, or segmental implies that the gene might arise from whole-genome duplication or segmental duplication.

### 3.4 Phylogenetic and syntenic analyses of *SbLLRLK* genes

**FIGURE 2:**
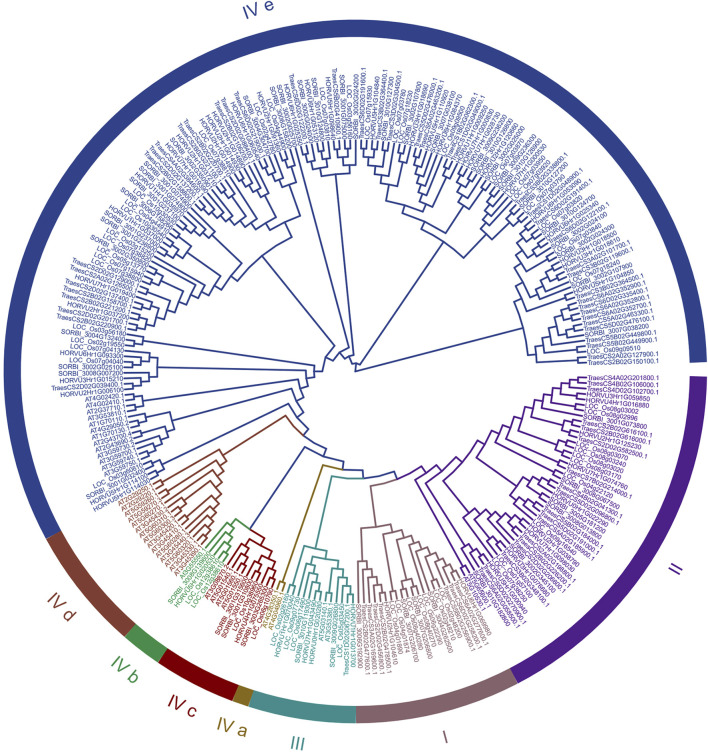
Evolutionary tree constructed based on the *LLRLK* family genes in sorghum and in other species. The neighbor-joining method of MEGA11 software was applied to construct the phylogenetic tree with 1,000 bootstrap replicates. The abbreviations used for different plant LLRLK proteins are as follows: At-*Arabidopsis thaliana*, SORBI-*Sorghum bicolor*, Os-*Oryza sativa*, Traes-*Triticum aestivum* and HORVU-*Hordeum vulgare*. Their functional differences are mainly reflected in the selection of the initial substrates and the number of condensation reactions.

The *SbLLRLK* proteins displayed a significant alignment with the *LLRLK* genes identified in *Arabidopsis*, rice, barley and wheat. They were also subdivided into four sub-groups based on the previous study (Vaid et al., 2013), with group IV being largest of them (and was sub-divided into five subgroups (Figure 2)). A total of 38, 72, 49 and 84 orthologs of the *SbLLRLK* genes were detected in *Arabidopsis*, rice, barley and wheat, respectively (Figure 2).

**FIGURE 3:**
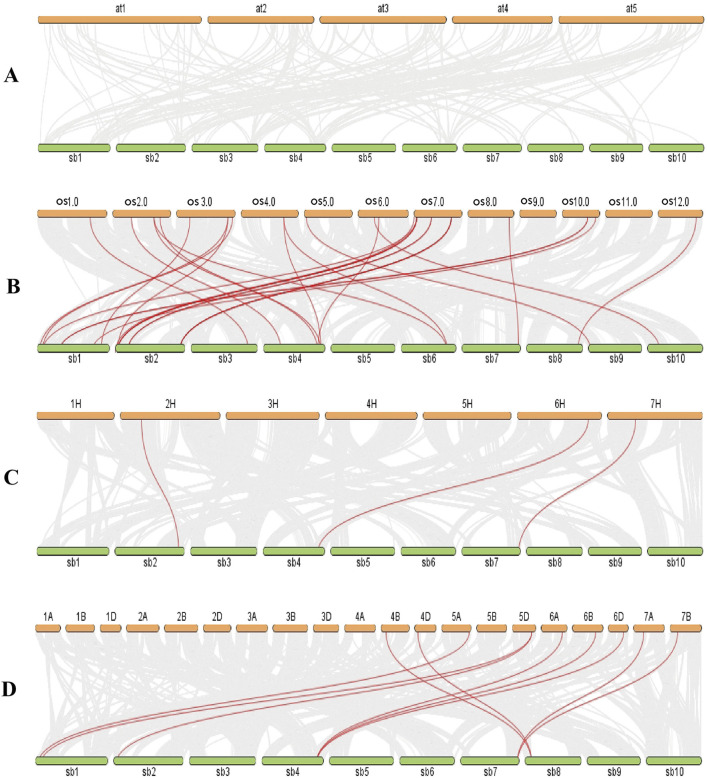
Synteny analysis for the *SbLLRLK* genes in sorghum and representative species such as **(A)** *Arabidopsis*, **(B)** rice, **(C)** barley and **(D)** wheat. The gray lines in the background represent the synteny regions in sorghum and in the genomes of other species, and the red lines represent the *SbLLRLK* gene pairs with synteny in sorghum and in other species. The abbreviations used for different plant chromosomes are as follows: at-*Arabidopsis thaliana*, sb-*Sorghum bicolor*, os-*Oryza sativa.* The gene pairs between sorghum and other species are listed in Supplementary Table S1, datasheet 2.

Most of the *SbLLRLKs* (32 out of 48) were classified together with rice, barley and wheat into Group I, II, III, and IV, while the *LLRLKs* from *Arabidopsis* were less distributed in these four groups. Group IV a and IV d contained *LLRLKs* only from *Arabidopsis*, while Group IV e contained the highest number of members of *LLRLKs* (Figure 2). In addition, the syntenic analysis of *LLRLK* genes in the different plant species showed that the *LLRLK* genes from sorghum presented no syntenic relationship with those in *Arabidopsis* (Figure 3A). However, the *LLRLK* genes from sorghum and rice showed a close syntenic relationship with each other. In total, 22 *SbLLRLK* members were found to be syntenic with the *LLRLKs* in rice (Figure 3B; Supplementary Table S1, datasheet 2). While the syntenic relationship of sorghum *LLRLK* genes with barley and wheat is not as pronounced as that observed with rice, there remains a notable degree of collinearity among these genes. Specifically, three *LLRLK* genes in sorghum exhibit a syntenic relationship with barley (Figure 3C; Supplementary Table S1, datasheet 2), while six such genes demonstrate a syntenic relationship with wheat (Figure 3D; Supplementary Table S1, datasheet 2).

### 3.5 Gene structure and protein motif analysis of the *SbLLRLKs*

**FIGURE 4:**
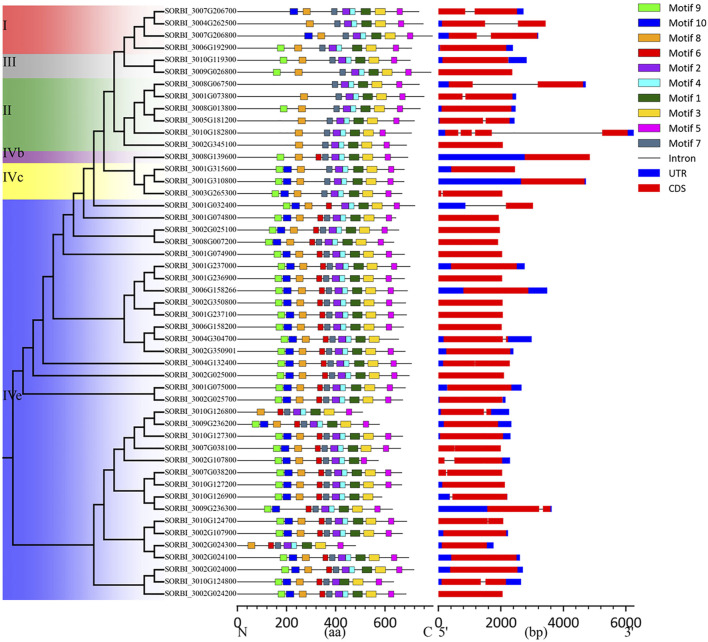
The conserved domains and gene structures of *SbLLRLK*. The distribution of the conserved domains is shown on the left, and the boxes with different colors represent different motifs. The scale bar at the bottom of the figure shows the gene length in the base pairs. The gene structure and conserved domains in the *SbLLRLK* family genes. The structural composition of the genes are shown on the right. The blue boxes represent the UTRs, the red boxes represent exons, and the dark lines connecting the two colored boxes represent introns.

To explore the structural diversity of *SbLLRLK* genes in sorghum, analysis of the exon-intron structure was conducted by comparing their coding sequences with the respective genomic sequences (Figure 4). The findings showed that *SbLLRLK* genes that were closely related tended to have comparable gene structures, including the same number of exons and introns (Figure 4). The differences primarily manifested in the varying lengths of UTRs and introns. Most *SbLLRLK* genes harbored a maximum of one intron, except for SORBI_3010G182800, which contained three introns. Furthermore, more than 60% (29 out of 48) of the *SbLLRLK* genes consisted of only a single exon (Figure 4).

Besides the difference in intron numbers, the length of introns also exhibited variability within *SbLLRLK* genes. Notably, SORBI_3008G067500, SORBI_3010G182800, and SORBI_3001G032400 displayed relatively larger introns compared to other members of the *SbLLRLK* genes (Figure 4). The variability in gene structure that has been documented indicates a potential influence on the evolution of the *SbLLRLK* gene family.

The MEME program was employed to identify conserved motifs in these *SbLLRLK* proteins. (Figure 4; Supplementary Table S1, datasheet 6). A total of 10 conserved motifs were identified, with the number of motifs ranging from 7 to 10 (Figure 4; Supplementary Table S1, datasheet 6). Despite the differences in motif numbers, the order of motifs 1–10 remained similar (Figure 4). Specifically, motif 5 was located at the C-terminus in all of the *SbLLRLK* proteins, while motif 10 was only found at the N-terminus. Approximately, the majority of *SbLLRLKs* (31 out of 49) contained all of the 10 motifs. In comparison, all of the *SbLLRLKs* contained motifs 1, 2, 3, 4, 5, and 7, which were annotated in the kinase domain, thus indicating that the kinase domain has remained conserved throughout the evolutionary process of sorghum. In the lectin domain, though a diversity in sequence was exhibited, there were still 3 conserved motifs (8, 9, and 10) in this region (Figure 4), indicating that the lectin domain was relatively unstable compared to the kinase domain during evolution.

### 3.6 Identification of the *SbLLRLK* gene associated with salt stress tolerance

A range of abiotic stresses and hormonal treatments were chosen in order to analyze the expression patterns of the *SbLLRLKs* in sorghum with different treatments. Gene expression levels were considered as upregulation when they showed a relative increase of more than two-fold, while downregulation was defined as a relative decrease of more than 0.5-fold in gene expression. An overview of the expressions of the different *SbLLRLK* genes in various abiotic stress conditions is shown in Supplementary Table S1, datasheet 4.

**FIGURE 5:**
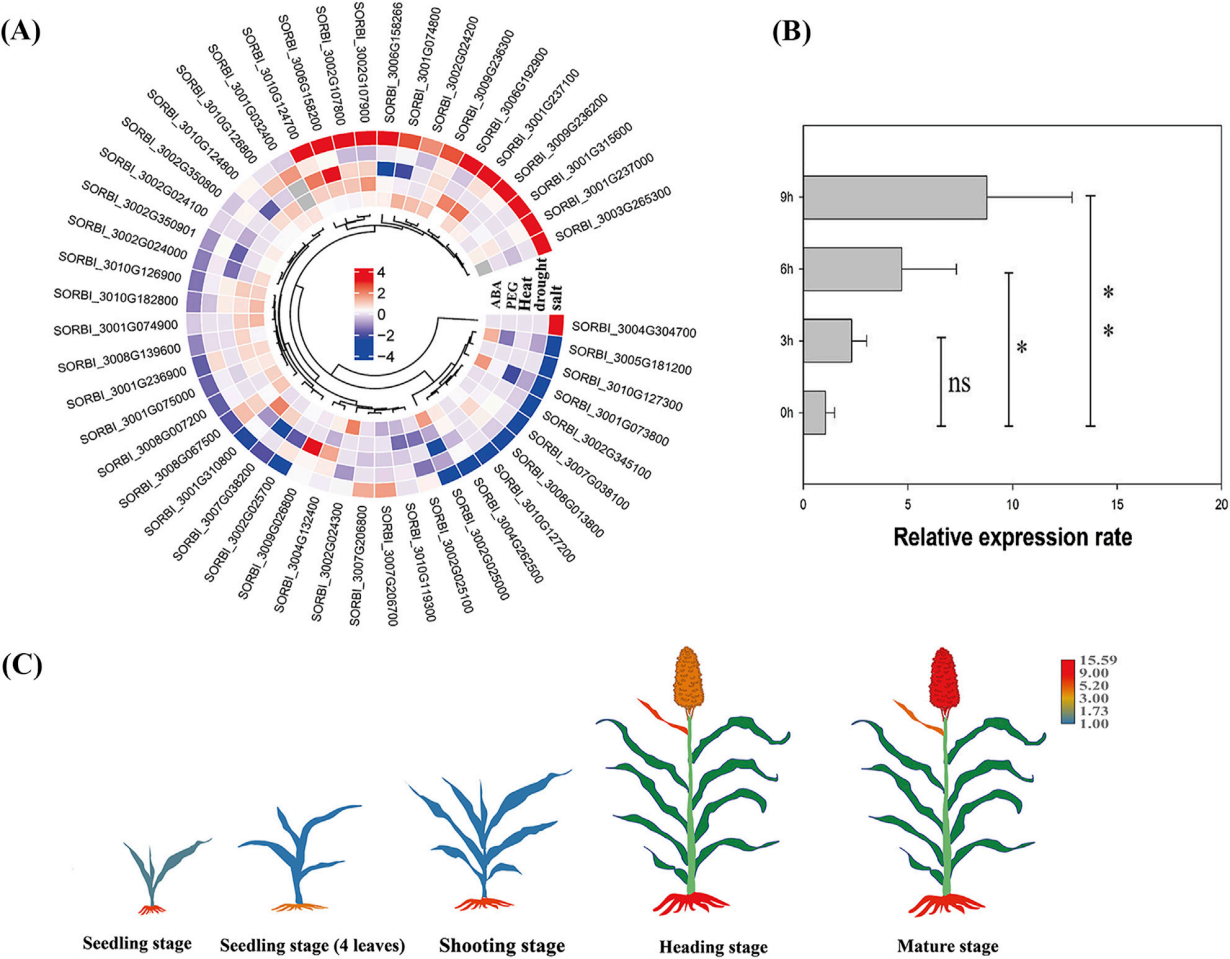
Expression profiles and function predication of the *SbLLRLK* genes in sorghum plants. **(A)** Expression pattern of *SbLLRLK* genes under different treatment. The change in the color of the box from blue to red indicates that the gene expression level changes from low to high. The grey cells represent no data. **(B)** qPCR analysis of SORBI_3004g304700 under 200 mM NaCl treatment. **(C)** Tissue-specific analysis of SORBI_3004g304700 at different stage of sorghum development. The change in the color of the different tissue from blue to red indicates that the gene expression level changes from low to high, while the green color indicates absence of expression.

Under salt stress conditions, approximately 70% (34 out of 48) of the *SbLLRLKs* exhibited desirable variations in their transcript levels. The number of *SbLLRLKs* exhibiting upregulation under salt stress condition was the same as that of the downregulated members (Figure 5A). Additionally, the *SbLLRLKs* in sorghum plants also exhibited different expression levels under the treatments of drought, PEG, heat, and ABA; however, the expression levels were not as significant as those found under salt-stress conditions (Figure 5A).

Specifically, after treatment with 200 mM NaCl for 6 h, the expression level of SORBI_3004g304700 significantly increased by nearly fourfold, but the expression rate showed no significance after 3 h of NaCl treatment (Figure 5B). After 9 h of treatment, the expression level increased up to 8-fold (Figure 5B). Moreover, the gene SORBI_3004g304700 was predominantly detected in the root tissues of sorghum plants from the early seedling stage to the mature stage (Figure 5C), suggesting a potential association with the plant’s ability to tolerate salt stress. Furthermore, SORBI_3004g304700 includes the initial nine out of ten motifs and contains only single short intron, which are typical features of *LLRLK* family. Therefore, SORBI_3004g304700 was chosen for further analysis.

### 3.7 Subcellular localization of SORBI_3004G304700

**FIGURE 6:**
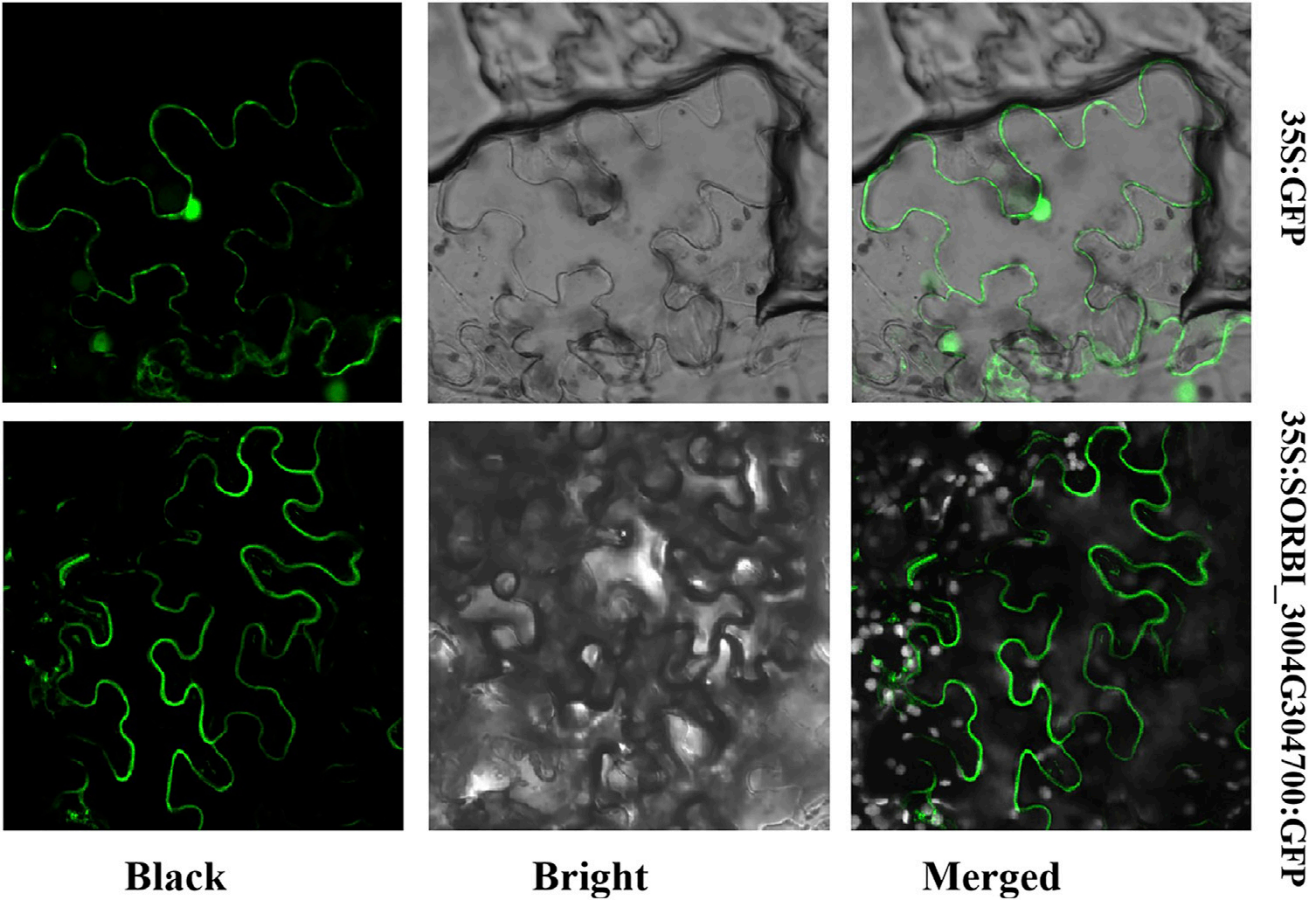
The subcellular localization of SORBI_3004G304700 in tobacco epidermal cells. SORBI_3004G304700 was fused to GFP at the C-terminal, which was transiently expressed in the tobacco epidermal cells. The instances of green fluorescence were observed with a confocal microscope.

We used an online tool, Cell-PLoc 2.0 (http://www.csbio.sjtu.edu.cn/bioinf/Cell-PLoc-2/), to predict the subcellular location (Supplementary Table S1, datasheet 1). SORBI_3004G304700 was predicted to be localized on the membrane. To confirm this prediction, SORBI_3004G304700 was fused to the PCEGFP vector (which harbors the 35S: SORBI_3004G304700: GFP expression cassette). The recombinant vector and the empty vector were introduced into the tobacco epidermal cells after 24 subculture with Agrobacterium. The results showed that the 35S: SORBI_3004G304700: GFP fusion protein is predominately located on the membrane of tobacco epidermal cells (Figure 6). However, in tobacco epidermal cells that are transformed with empty vectors, green fluorescence could be detected in all parts of the cells (Figure 6). The results suggest that SORBI_3004G304700 is a protein located on the cell membrane and that it may be involved in signal transduction.

### 3.8 The functional validation of SORBI_3004G304700 as a negative modulator of salt stress tolerance

**FIGURE 7:**
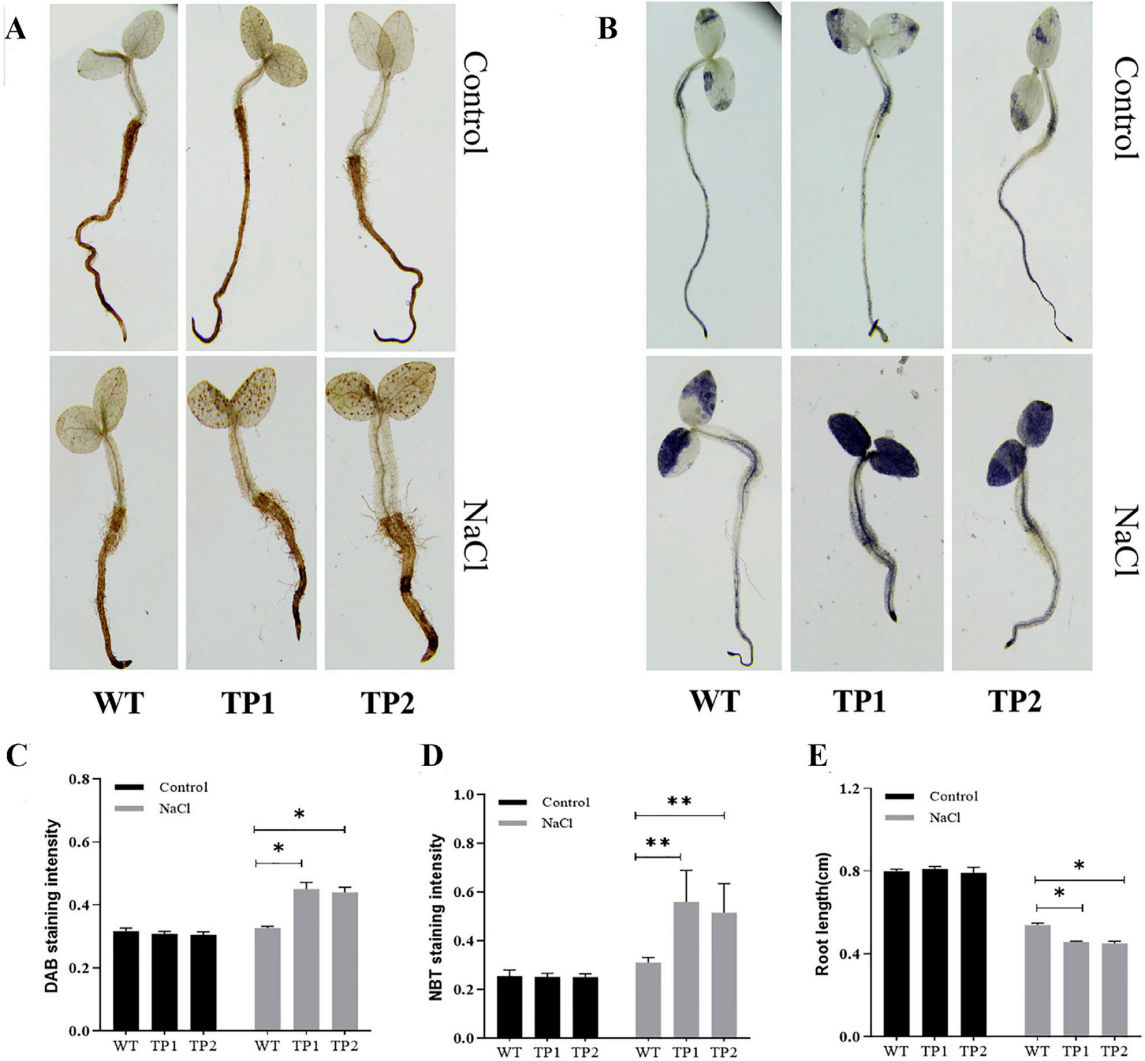
Function validation of SORBI_3004G304700 by tobacco transformation. DAB staining intensity of **(A)** wild-type (WT) tobacco and **(B)** transgenic tobacco lines (TPs). NBT staining intensity of **(C)** wild-type (WT) tobacco and **(D)** transgenic tobacco (TPs) lines. **(E)** The root length analysis of wild-type tobacco (WT) and transgenic tobacco lines (TPs). The abbreviations are listed as follows: WT-wild type plants, TP1 and TP2 - transgenic plants, DAB -3,3′-Diaminobenzidine and NBT-nitrobluetetrazolium.* and ** indicate the values between groups that reached the significance levels of *P* < 0.05 and *P* < 0.01, respectively.

In order to further confirm the functionality of SORBI_3004G304700, over-expression experiments were conducted in transgenic tobacco plants. Two transgenic plants (TP1 and TP2) were subsequently obtained for detailed analysis (Figure 7). Both the transgenic plants and wild-type tobacco plants were exposed to salt-stress conditions (200 mM NaCl) as well as normal conditions (0 mM NaCl) to assess their resistance to salt stress. The accumulation of H_2_O_2_ and superoxide in salinity-stressed tobacco seedlings was evaluated using 3, 3′-diaminobenzidine (DAB) staining and nitrobluetetrazolium (NBT) staining. The findings revealed that the levels of NBT and DAB staining were similar between the transgenic plants (TP1 and TP2) and wild-type plants under normal circumstances. However, when exposed to salt stress, the transgenic plants exhibited significantly higher intensities of DAB and NBT staining compared to the wild-type plants (Figures 7A–D), indicating increased production of H_2_O_2_ and reactive oxygen species (ROS) in the transgenic plants under salt-stress conditions. Furthermore, the root length of the transgenic plants was notably shorter than that of the wild-type plants following NaCl treatment (Figure 7E), suggesting a diminished tolerance to salt stress in the transgenic plants. Consequently, it can be inferred that SORBI_3004G304700 acts as a suppressor of salt stress tolerance.

### 3.9 Gene variation and haplotype analysis of SORBI_3004G304700

**FIGURE 8:**
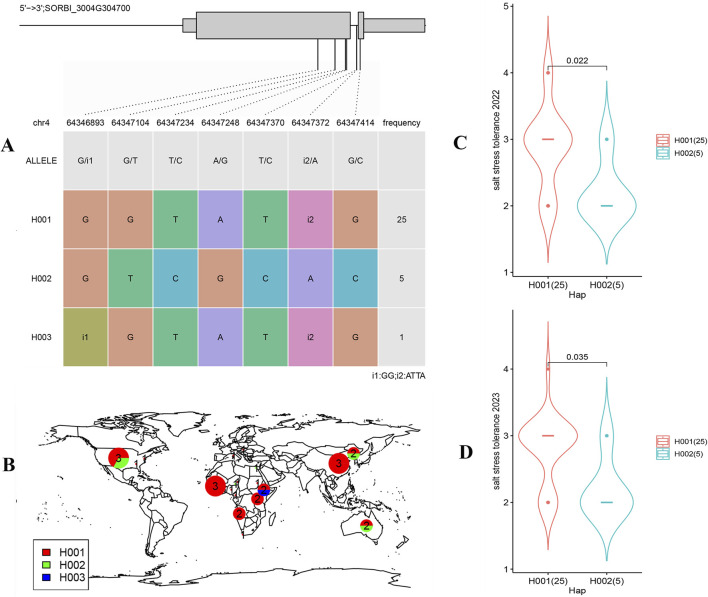
The haplotypes analysis of the SORBI_3004G304700. **(A)** SNPs identified for haplotype analysis of SORBI_3004G304700. The wide boxes indicate exons and the narrow boxes indicated UTRs. The straight line between the boxes indicate introns, and SNPs in the coding sequence of the gene are shown in the lower table. **(B)** Geographical distribution of different haplotypes. H001-H003 were marked in different colors. **(C, D)** The correlation analysis of haplotypes with salt stress tolerance in two different years. Statistical significance was determined by Student’s t-test. The material numbers used for statistical analyses are shown under each haplotype.

The haplotypes of the SORBI_3004G304700 were analyzed to investigate its vsariation. Within 31 sorghum cultivars, 3 unique haplotypes (H001-H003) and 7 different sites of variation were identified in the SORBI_3004G304700 gene (Figure 8A). H001 emerged as the most prevalent haplotype, while H003 was observed in only one cultivar (Figure 8A). H003 exhibits similar variations type to H001, with the exception of an indel located at position 64,346,893. The variant sites of H002 differ significantly from those of haplotypes 1 and 3 (Figure 8A). Furthermore, there are distinct single nucleotide polymorphisms (SNP) and indel insertions in the introns of the SORBI_3004G304700 gene that distinguish haplotype 2 from haplotypes 1 and 3 (Figure 8A). Notably, the number of sorghum cultivars containing H002 (5 out of 31) is lower compared to those containing H001 (Figure 8A). Additionally, H001 is prevalent across the worldwide distribution of sorghum (Figure 8B), suggesting that this haplotype may have undergone favorable selection pressures during the cultivation of sorghum. Haplotype analysis revealed that H001 exhibited greater resilience to salt stress in both 2022 and 2023 when compared to H002 (Figures 8C, D).

## 4 Discussion

*LLRLKs* are widely present in all higher plants. Notably, *LLRLKs* harbor an extracellular domain resembling lectin proteins (Shiu and Bleecker, 2001). Extensive investigations have been conducted on the functions and regulation of *LLRLK* genes in various plants. For example, *Arabidopsis* and rice have been reported to harbor 38 and 72 *LLRLK* genes, respectively (Figure 2) (Vaid et al., 2012). They have been associated with a wide range of physiological functions, including protein binding, organic growth, and defense against different types of stressors. (Sun et al., 2020; Singh et al., 2021). Limited information has been gathered about the LLRLK family in sorghum, which hinders our comprehension of the molecular mechanisms associated with LLRLKs in this particular species. Therefore, a genome-wide analysis was conducted and 49 *SbLLRLKs* were identified in sorghum (Figure 1).

The number of *LLRLK* gene family members varies in different plants. For example, wheat variety such as “Chinese Spring” has a genome of more than 14 GB (Zhu et al., 2021), and it contains 83 *LLRLK* genes (Sharma et al., 2016).In contrast, the sorghum plant, with its relatively small genome size of less than 1 GB (Ananda et al., 2020),exhibits a lower number of *LLRLK* genes (49). This may be attributed to the variation in genome sizes of different plant species, which can influence the size of gene families (Geng et al., 2022). In addition, tandem duplication and segmental duplication are important mechanisms contributing to gene family expansion during genome evolution, thus impacting the number of *LLRLK* genes. Our examination of gene duplication events revealed that the 49 *SbLLRLK* genes contained only 5 duplicated genes (Figure 1), which was fewer than that found in other plants such as rice (Lannoo and Van Damme, 2014). The lower occurrence of tandem duplication events in sorghum may account for a smaller number of *SbLLRLK* genes.

Structural composition is a fundamental aspect of gene function, as it determines the gene’s ability to interact with other cellular components and carry out its intended role (Parenteau et al., 2019). The evolutionary dynamics of gene families involve the gain and loss of introns (Carmel et al., 2012). In higher plants, introns serve as a common feature of genes (Oudelaar and Higgs, 2021; Chen et al., 2022). Their regulatory role in gene expression is multifaceted, they can impact transcription levels through modulating transcription rates, nuclear output and transcriptional stability (Jiao et al., 2007).

The gene structure analysis suggested that the whole *SbLLRLK* gene family is rather intronless, with only 17 genes out of 49 having intronic regions (Figure 4). The other 31 of the *SbLLRLK* genes contained a single exon, accounting for more than 60% of the total *SbLLRLK* genes (Figure 4). Moreover, 16 out of 17 genes with intron regions contained only one intron (Figure 4). Previous research on *Arabidopsis* and rice have similarly indicated the absence of introns within the *LLRLK* gene family (Bellande et al., 2017). For example, only 5 and 8 genes within *Arabidopsis* (38 *LLRLK* genes) and rice (72 *LLRLK* genes) have introns, respectively (Vaid et al., 2012; Sun et al., 2020). The lack of introns in these genes may be attributed to their role as signal receptors in plants. The compact gene architecture is thought to improve the effectiveness of gene expression by reducing alternative splicing and preserving energy, especially for genes responsible for reacting to various environmental cues.

By analyzing the protein sequences of *SbLLRLKs*, we found that the DFGL located between β8 and β9 and the threonine (T) and phenylalanine (F) at the position of 361 and 364 are highly conserved in the kinase domains (Supplementary Figure S2), which is consistent with the previous research in other species (Cavada et al., 2020). Moreover, GxGxxG motif has been implicated as a nucleotide binding site and considered as the active position of *LLRLK* genes which have been demonstrated in other species (Zhu et al., 2021). Additionally, the number of conserved motifs is also stable in the kinase domain (Figure 4). This indicated that the kinase domain is structurally and functionally conservative during the evolution of sorghum.

Compared with the kinase domain, the amino acid sequence of lectin domain exhibited considerable variability. There is only a conserved glycine (G) at the position of 253 in the lectin domain (Supplementary Figure S1). The Variations in lectin domain are also detected in other species such as *Arabidopsis* and rice. This observation suggests that functional divergence during evolution process can be resulted from mutations in nonconserved amino acids within the lectin domain.

Duplication events serve as a driving force for generating new genes, while functional differentiation acts as a catalyst for their emergence (Assis and Bachtrog, 2015; Kang et al., 2024). To evaluate the selection pressures on *SbLLRLKs*, the Ka/Ks ratios for the 5 duplicated *SbLLRLK* genes were calculated. The Ka/Ks ratios presented in Table 1 are all below 1, indicating that these duplicated *SbLLRLKs* have experienced significant selection constraints during evolution. In addition, the syntenic analysis of the *SbLLRLK* genes in different plants showed that the numbers of syntenic *SbLLRLK* pairs between sorghum and monocot (rice, barley and wheat) were more than those between sorghum and other dicots (*Arabidopsis*) (Figure 3). This observation suggests a closer syntenic relationship of *SbLLRLKs* with monocots rather than with dicots. Moreover, the *SbLLRLK* genes demonstrate a more proximate phylogenetic relationship with *OsLLRLK* in comparison to *HvLLRLK* and *TaLLRLK* (Figure 3). This finding may be explained by differences in evolutionary distance and divergence time among the respective species (Zhang et al., 2024).

The functions of genes can be inferred through the examination of their sequence homology. In the present study, a collinear analysis detected 22 linear orthologous genes of *SbLLRLKs* in rice (Supplementary Table S1, datasheet 2).This is especially relevant for the homologous gene SORBI_3004G304700 in rice, designated as Os02g0640500 (LOC_Os02g42780), which has been demonstrated to function as a negative regulator of salt stress tolerance in rice. This gene has been subsequently renamed *SIT1* (salt intolerance 1) (Li et al., 2014) (Supplementary Table S1, datasheet 2). Additionally, the homologous genes of *SIT2* (LOC_Os04g44900) is SORBI_3006G158200. Those type of genes share very similar structures (Supplementary Figure S3) and are clustered into the same secondary clade of a phylogenetic tree (Figure 2).This indicated that the two pairs of genes may play the same role in salt stress tolerance within different species.

The transcriptome analysis of hormone and stress treatments revealed that a majority of *SbLLRLKs* (31 out of 49) exhibited significant responses to at least one treatment (fold-change >1) (Figure 5A). Uniquely, the expression level of SORBI_3004G304700 were changed obviously after salt treatment and it were founded mainly expressing in the root of sorghum (Figures 5B, C). These results strongly support the prediction that the orthologous gene of rice *SIT1* (Li et al., 2014) in sorghum, i.e., SORBI_3004G304700, is significantly associated with plant resistance to salt stress. Syntenic analysis indicate that SORBI_3004G304700 share collinearity with *OsSIT1* (Supplementary Table S1, datasheet 2), which was also demonstrated to be localized on the membrane (Figure 6) and considerd as a gene associated with salt resistance (Li et al., 2014).

To test this presumption, we used transgenic tobacco plants to further investigate the function of this gene. Based on previous reports, staining methods such as DAB and NBT have been proven to be effective in detecting the accumulation of reactive oxygen species (ROS) in different plant materials (Li et al., 2014) (Figure 7). Specifically, we observed a significant increase in the staining intensity of two transgenic lines when compared to wild-type materials, indicating a higher level of ROS accumulation in these strains (Figures 7A–D). In addition, the transgenic plants (TPs) exhibited significantly reduction of root length compared to the wild-type plants when subjected to NaCl treatment, indicating a reduction in salt stress (Figure 7E). This observation is consistent with previous studies that have reported increased ROS accumulation and reduced salt-stress ability in transgenic plants that overexpress *OsSIT1* (Li et al., 2014) (which is the putative homologous gene of SORBI_3004g304700 and which acts as a negative regulator of plant resistance to salt stress). In sorghum, SORBI_3004g304700 is considered the homologous gene of *OsSIT1* (LOC_Os02g42780) (Figure 2; Supplementary Table S1, datasheet 2). Therefore, the reduction in salt stress in TPs may be as a result of the overexpression of SORBI_3004g304700 and its activation of the same stress response pathways as *OsSIT1*.

The *GmLLRLK* gene from soybean has been shown to enhance the scavenging of reactive oxygen species (ROS) in soybean plants when subjected to salt stress (Wang et al., 2021). This enhancement in ROS scavenging capacity suggests a protective role for the *GmLLRLK* gene under saline conditions. Furthermore, many other research has demonstrated that overexpression of the *GmLLRLK* gene in soybean hairy roots alone is sufficient to confer salt tolerance (Sun et al., 2018). These results highlighting the importance of *GmLLRLK* in the adaptive strategies of soybean to saline conditions. In cereal crops such as barley and wheat, the *LLRLK* gene, is essential for the regulation of salt tolerance (Ahmed et al., 2024). These genes significantly affects seed germination rates and the survival of seedlings in saline conditions. Genetic modification of plants to overexpress the some *LLRLK* genes results in a decreased accumulation of salt ions (Yan et al., 2023). Additionally, these modified plants exhibited increased capacity to produce compounds that alleviate the impacts of salt stress, including proline and malondialdehyde (MDA) (Yan et al., 2023). This enhanced capability to cope with salt stress is vital for the resilience of these crops in saline environments.

Numerous studies have shown that *LLRLK* genes can activate the MAPK3/6 signaling cascade through direct phosphorylation, which subsequently influences downstream signal transduction (Vaid et al., 2013). This activation ultimately affects various hormone-related metabolic pathways and the clearance of reactive oxygen species (ROS), thereby playing a significant role in salt tolerance (Bellande et al., 2017). Moreover, *LLRLK* can also affects certain transcription factors related to the synthesis of ethylene and ABA by protein-protein interactions, thereby influencing salt tolerance (Li et al., 2014). In this study, we identified one of the *SbLLRLK* gene as a regulator of salt stress tolerance via modulating ROS accumulation, which demonstrated the potential of this gene family in salt stress resistance. However, the specific molecular mechanism of *LLRLK* requires further research.

Based on the expression pattern of SORBI_3004G304700 combined with the reported functional study of their homologous gene (Figure 5), different haplotypes associated with salt stress tolerance of sorghum were identified. For example, only a single cultivar containing H003 was detected and its variation sites were almost the same as that of H001 except for the insertion at the location of 64,346,893 which can cause the frame shift mutation (Figure 8A). Furthermore, this variation site had never been reported. Therefore, this haplotype may be caused by sequencing or assembly errors. In comparison, H002 and H001 are two distinct haplotypes that exhibit significant differences in their mutation sites (Figure 8A). These mutations may confer different traits and responses to salt stress. The salt stress resistance analysis conducted over two consecutive years (2022–2023) has provided valuable insights into the performance of varieties containing the H002 haplotype compared to H001 (Figures 8C, D). The findings suggest that the H002 haplotype may be less adapted to saline conditions (Figures 8C, D), indicating a potential area for genetic improvement. The geographical distribution analysis of H001 and H002 haplotypes shows a predominance of H001 (Figure 8A), suggesting its widespread adaptation and possibly superior performance in various environmental conditions (Figure 8B).

The development of molecular markers for the enhancement of breeding programs can be achieved through the identification of Single Nucleotide Polymorphisms (SNPs) or Insertions/Deletions (InDels) within the genetic variations observed in our study (Bhat et al., 2021; Dai et al., 2024). In a previous work, many markers have been employed to facilitate the cultivation of soybean varieties with elevated protein levels (Hwang et al., 2014). Additionally, DNA markers have been instrumental in pinpointing genotypes that exhibit enhanced abiotic resistance and increased barley yields. In this study, Hap001 of SORBI_3004g304700 was the superior haplotype contributing to salt stress tolerance, which could be selected by detecting InDels (i2) in Hap001 to improve breeding efficiency of sorghum. This information is instrumental for designing molecular markers and developing assisted selection breeding strategies that can leverage the genetic advantages of H001.

Our study has characterized the SORBI_3004g304700 gene as a pivotal negative regulator in sorghum’s salt stress response. This insight is crucial for the future breeding of salt-resistant crops, which could significantly elevate agricultural output in regions where soil salinization is prevalent. However, further research is needed to explore the molecular mechanisms by which SORBI_3004g304700 regulates salt stress tolerance and to explore its potential as a target for genetic engineering in the context of crop improvement.

## 5 Conclusion

In this study, we employed bioinformatics techniques to identify a total of 49 *SbLLRLK* genes in sorghum, with the majority being intron-less. These genes were phylogenetically categorized into four distinct subfamilies. RNA-seq analysis revealed that each member of the *SbLLRLK* gene family displayed a unique expression profile in response to hormonal and stress stimuli. Furthermore, through comparative gene analysis and subsequent RNA-seq validation, we identified SORBI_3004g304700 as a candidate regulator for salt stress tolerance. Haplotype analysis identified three distinct haplotypes (H001-H003) of the SORBI_3004G304700 gene. Notably, sorghum cultivars possessing the H001 haplotype demonstrated enhanced salt stress tolerance and a broader geographical range. The results of this study not only provide a thorough characterization of the *SbLLRLK* gene family in sorghum but also contribute significant insights into their potential role in conferring salt-stress resistance.

## Data Availability

The datasets presented in this study can be found in online repositories. The names of the repositories and accession number(s) can be found in the article/Supplementary Material.

## Supplementary material

SUPPLEMENTARY FIGURE S1 The lectin domains in the 49 *SbLLRLK* genes.

SUPPLEMENTARY FIGURE S2 The kinase domains in the 49 *SbLLRLK* genes.

SUPPLEMENTARY FIGURE S3 3D structure of kinase domain from **(A)** SORBI_3004G304700; **(B)** SORBI_3006G158200; **(C)** SORBI_3001G074900 and **(D)** SORBI_3002G024000.

SUPPLEMENTARY FIGURE S4 Gene cloning confirmation in vector.

SUPPLEMENTARY FIGURE S5 The cloning strategy in this study.

SUPPLEMENTARY FIGURE S6 Transgenic tobacco development steps.

SUPPLEMENTARY FIGURE S7 PCR and QPCR confirmation of transgenic tobacco lines.

SUPPLEMENTARY TABLE S1 The research data related to this study.
