# Supplementary material for: Identification, function validation and haplotype analysis of salt-tolerant genes of lectin receptor kinase gene family in sorghum (Sorghum bicolor L.)
Source: Front Genet. 2024 Oct 15;15:1464537. doi: 10.3389/fgene.2024.1464537 (PMC11518778; doi:10.3389/fgene.2024.1464537)
Supplement: Supplementary file 1 [file DataSheet7.PDF]

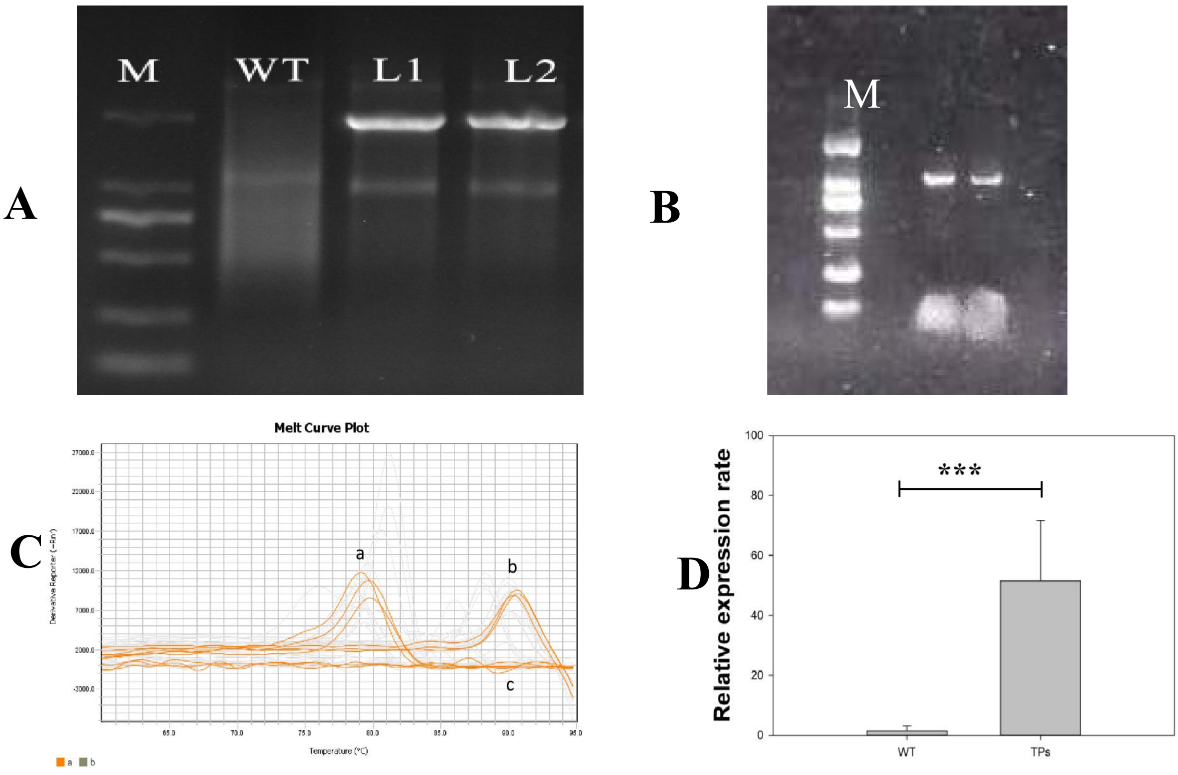

Supplementary Figure 7: PCR and QPCR confirmation of transgenic tobacco lines. (A) L1 and L2 are lanes for TP1 and TP2 respectively and the marker is DL2000. the target PCR product is 1926bp.(B) the plant selection marker(HYG) PCR confirmation and the target product is 1026bp.The marker is DL2000 (C) the melt curve for QRT-PCR. curve a indicate the Nbactin and curve b indicate the QRT-PCR for SORBI_3004g304700 in the TPs, while curve c indicated the QRT-PCR for SORBI_3004g304700 in the WT plants.(D)QRT-PCR confirmation of WT and TPs.*** represent the 0.001 significance difference between the WT and TPs
