# Supplementary material for: Identification, function validation and haplotype analysis of salt-tolerant genes of lectin receptor kinase gene family in sorghum (Sorghum bicolor L.)
Source: Front Genet. 2024 Oct 15;15:1464537. doi: 10.3389/fgene.2024.1464537 (PMC11518778; doi:10.3389/fgene.2024.1464537)
Supplement: Supplementary file 2 [file DataSheet2.PDF]

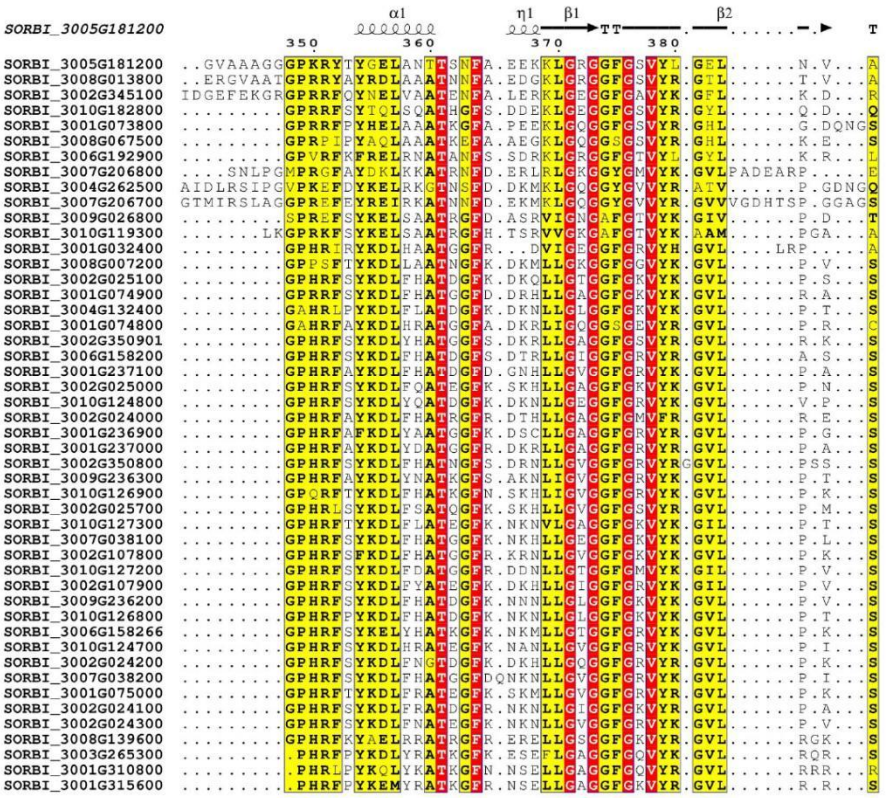
SORBI_3005G181200
α1
η1
β1
β2
350
360
370
380
SORBI_3005G181200 ..GVAAAGGGPKRYTYGELANTTSNFA.EEKKLGRGGFGSVYL.GEL.....N.V...A
SORBI_3008G013800 ..ERGVAATGPRRYAYRDLAAATNNFA.EDGKLGRGGFGSVYR.GTL.....T.V...A
SORBI_3002G345100 IDGEFEKGRGPRRFQYNELVAATENFA.LERKLGEGGFGAVYK.GFL.....K.D...R
SORBI_3010G182800 .........GPRRFSYTQLSQATHGFS.DDEKLGEGGFGSVYR.GYL.....Q.D...Q
SORBI_3001G073800 .........GPRRFPYHELAAATKGFA.PEEKLGQGGFGSVYR.GHL.....G.DQNGS
SORBI_3008G067500 .........GPRRPIPYAQLAAATKEFA.AEGKLGQGGSGSVYR.GHL.....K.E...S
SORBI_3006G192900 .........GPVRFKFRELRNATANFS.SDRKLGRGGFGTVYL.GYL.....K.R...L
SORBI_3007G206800 ....SNLPGMPRGFAYDKLKKATRNFD.ERLRLGKGGYGMVYK.GVLPADEARP.....E
SORBI_3004G262500 AIDLRSIPGVPKEFDYKELRKGTNSFD.DKMKLGQGGYGVVYR.AIV.....P.GDNGQ
SORBI_3007G206700 GTMIRSLAGGPREFEYREIRKATNNFD.EKMKLGQGGYGVVYR.GVVVGDHTSP.GGAGS
SORBI_3009G026800 .........SPREFSYKELSAATRGFD.ASRVIGNGAFGTVYK.GIV.....P.D...T
SORBI_3010G119300 .......LKGPRKFSYKELSAATRGFH.TSRVVGKGAFGTVYK.AAM.....PGA...A
SORBI_3001G032400 .........GPHRIRYKDLHAATGGFR...DVIGEGGFGRVYH.GVL....LRP.....A
SORBI_3008G007200 .........GPPSFTYKDLLAATNGFK.DKMLLGKGGFGGVYK.GVL.....P.V...S
SORBI_3002G025100 .........GPHRFSYKDLFHATDGFK.DKQLLGTGGFGKVYK.GVL.....P.S...S
SORBI_3001G074900 .........GPRRFSYKDLFHATGGFD.DRHLLGAGGFGKVYK.GVL.....R.A...S
SORBI_3004G132400 .........GAHRLPYKDLFLATDGFK.DKNLLGIGGFGKVYK.GVL.....P.T...S
SORBI_3001G074800 .........GAHRFAYKDLHRATGGFA.DKRLIGQGGSGRVYR.GVL.....P.R...C
SORBI_3002G350901 .........GPHRFSYKDLYHATGGFS.DKRLLGAGGFGSVYR.GVL.....R.K...S
SORBI_3006G158200 .........GPHRFSYKDLFHATDGFS.DTRLLGIGGFGRVYR.GVL.....A.S...S
SORBI_3001G237100 .........GPHRFAYKDLFHATDGFD.GNHLLGVGGFGRVYK.GVL.....P.A...S
SORBI_3002G025000 .........GPHRFSYKDLFQATEGFK.SKHLLGAGGFGKVYK.GVL.....P.N...S
SORBI_3010G124800 .........GPHRFSYKDLYQATDGFK.DKNLLGEGGFGRVYK.GVL.....V.P...S
SORBI_3002G024000 .........GPHRFAYKDLFHATRGFR.DTHLLGAGGFGMVFR.GVL.....R.E...S
SORBI_3001G236900 .........GPHRFAFKDLYAATGGFK.DSCLLGAGGFGRVYR.GVL.....P.G...S
SORBI_3001G237000 .........GPHRFAYKDLYDATGGFR.DKRLLGAGGFGRVYR.GVL.....P.A...S
SORBI_3002G350800 .........GPHRFSYKDLFHATNGFS.DRNLLGVGGFGRVYRGGVL.....PSS...S
SORBI_3009G236300 .........GPHRFAYKDLYNATKGFS.AKNLIGVGGFGRVYK.GVL.....P.T...S
SORBI_3010G126900 .........GPQRFTYKDLFHATKGFN.SKHLIGVGGFGRVYK.GVL.....P.K...S
SORBI_3002G025700 .........GPHRLSYKDLFSATQGFK.SKHLLGVGGFGSVYR.GVL.....P.M...S
SORBI_3010G127300 .........GPHRFTYKDLFLATEGFK.NKNVLGAGGFGKVYK.GIL.....P.T...S
SORBI_3007G038100 .........GPHRFSYKDLFHATGGFK.NKHLLGEGGFGKVYK.GVL.....P.L...S
SORBI_3002G107800 .........GPHRFSFKDLFHATGGFR.KRNLLGVGGFGKVYK.GVL.....P.K...S
SORBI_3010G127200 .........GPHRFSYKDLFDATGGFR.DDNLLGTGGFGMVYK.GIL.....P.V...S
SORBI_3002G107900 .........GPHRFSYKDLFYATEGFK.DKHLLGIGGFGRVYK.GIL.....P.V...S
SORBI_3009G236200 .........GPHRFSYKDLFHATDGFK.NNNLLGLGGFGKVYK.GVL.....P.V...S
SORBI_3010G126800 .........GPHRFPYKDLFHATDGFK.NKNLLGIGGFGKVYK.GVL.....P.T...S
SORBI_3006G158266 .........GPHRFSYKELYHATKGFK.NKMLLGTGGFGRVYK.GVL.....P.K...S
SORBI_3010G124700 .........GPHRFSYKDLHRATEGFK.NANLLGVGGFGRVYK.GVL.....P.I...S
SORBI_3002G024200 .........GPHRFSYKDLFNGTDGFK.DKHLLGQGGFGRVYR.GVL.....P.K...S
SORBI_3007G038200 .........GPHRFSYKDLFHATGGFDQNKNLLGVGGFGRVYK.GVL.....P.I...S
SORBI_3001G075000 .........GPHRFTYKDLFRATEGFK.SKMLLGVGGFGRVYR.GVL.....P.K...S
SORBI_3002G024100 .........GPHRFSYKDLFRATDGFR.NKNLLGIGGFGKVYR.GVL.....P.A...S
SORBI_3002G024300 .........GPHRFSYKDLFNATEGFK.NKNLLGVGGFGRVYK.GVL.....P.V...S
SORBI_3008G139600 .........GPHRFKYAELRRATRGFR.ERELLGSGGFGKVYR.GVL.....RGK...S
SORBI_3003G265300 .........PHRFPYKDLYRATKGFK.ESEFLGAGGFGQVYK.GVL.....RQR...S
SORBI_3001G310800 .........PHRLPYKQLYKATKGFN.NSELLGAGGFGQVYR.GVL.....RRR...R
SORBI_3001G315600 .........PHRFPYKEMYRATKGFR.NSELLGAGGFGKVYR.GVL.....R.R...S

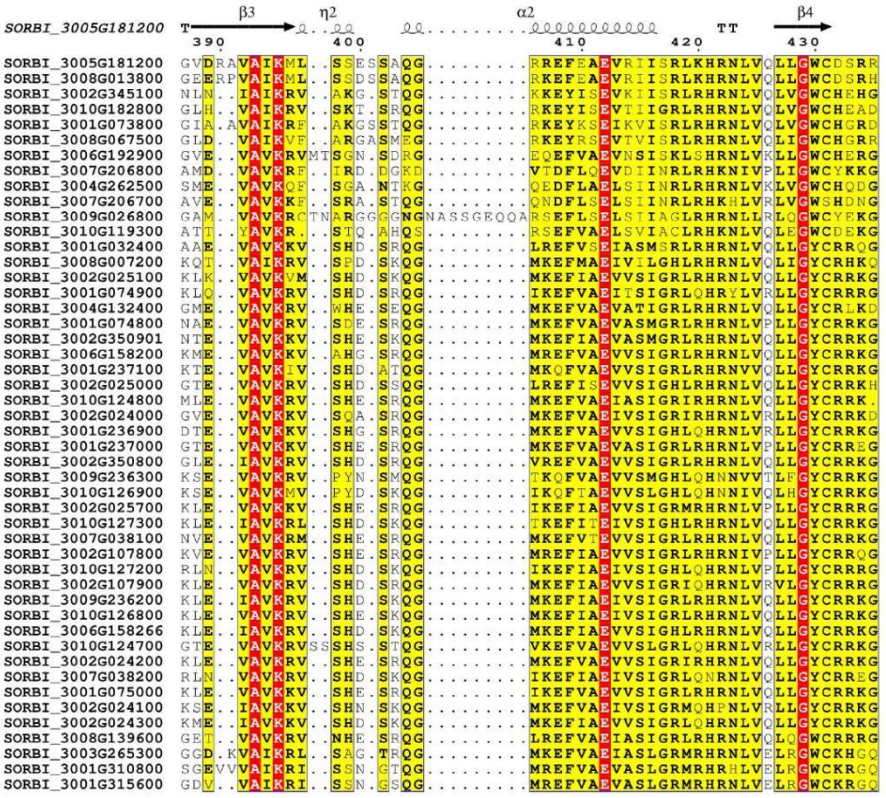
SORBI_3005G181200
β3
η2
α2
β4
390
400
410
420
430
SORBI_3005G181200 GVDRAVAIKMLSSESSAQG.........RREFEAEVRIISRLKHRNLVQLLGWCDSRR
SORBI_3008G013800 GEERPVAIKMLSSDSSAQG.........RKEFEAEVRIISRLKHRNLVQLLGWCDSRH
SORBI_3002G345100 NLN..IAIKRVAKG.STQG.........KKEYISEVKIISRLRHRNLVQLVGWCHEHG
SORBI_3010G182800 GLH..VAIKRVSKT.SRQG.........RKEYISEVTIIGRLRHRNLVQLVGWCHEAD
SORBI_3001G073800 GIA.AVAIKRFAKGSSTQG.........RKEYKSEIKVISRLRHRNLVQLVGWCHGRD
SORBI_3008G067500 GLD..VAIKVFARGASMEG.........RKEYRSEVTVISRLRHRNLVQLIGWCHGRR
SORBI_3006G192900 GVE..VAVKRVMTSGN.SDRG.........EQEFVAEVNSISKLSHRNLVKLLGWCHERG
SORBI_3007G206800 AMD..VAVKRFIRD.DGKD.........VTDFLQEVDIINRLRHKNIVPLIGWCYKKG
SORBI_3004G262500 SME..VAVKQFSGA.NTKG.........QEDFLAELSIINRLRHRNLVKLVGWCHQDG
SORBI_3007G206700 AVE..VAVKKFSRA.STQG.........QNDFLSELSIINRLRHKHLVRLVGWSHDNG
SORBI_3009G026800 GAM..VAVKRCTNARGGGGNGNASSGEQQARSEFLSELSIAGLRHRNLLRLQGWCYFKG
SORBI_3010G119300 ATT..YAVKRSTQ.AHQS.........RSEFVAELSVIACLRHKNLVQLEGWCDEKG
SORBI_3001G032400 AAE..VAVKKVSHD.SRQG.........LREFVSEIASMSRLRHRNLVQLLGYCRRQG
SORBI_3008G007200 KQT..VAIKRVSPD.SKQG.........MKEFMAEIVILGHLRHRNLVQLIGYCRHKQ
SORBI_3002G025100 KLK..VAVKVMSHD.SKQG.........MKEFIAEVVSIGRLRHRNLVQLLGYCRRKG
SORBI_3001G074900 KLQ..VAVKRVSHD.SRQG.........IKEFVAEITSIGRLQHRYLVRLLGYCRRRG
SORBI_3004G132400 GME..VAVKRVWHE.SEQG.........MKEFVAEVATIGRLRHRNLVQLLGYCRLKD
SORBI_3001G074800 NAE..VAVKRVSDE.SRQG.........MKEFVAEVASMGRLRHRNLVPLLGYCRRKG
SORBI_3002G350901 NTE..VAVKKVSHE.SKQG.........MKEFIAEVASMGRLRHRNLVQLLGYCRRKG
SORBI_3006G158200 KME..VAVKRVAHG.SRQG.........MREFVAEVVSIGRLRHRNLVQLLGYCRRKG
SORBI_3001G237100 KTE..VAVKIVSHD.ATQG.........MKQFVAEVVSIGRLRHRNVVQLLGYCRRKG
SORBI_3002G025000 GTE..VAVKRVSHD.SSQG.........LREFISEVVSIGHLRHRNLVQLLGYCRRKH
SORBI_3010G124800 MLE..VAVKRVSHE.SRQG.........MKEFVAEIASIGRIRHRNLVQLLGYCRRK.
SORBI_3002G024000 GVE..VAVKKVSQA.SRQG.........MKEFVAEIVSIGRIRHRNLVRLLGYCRRKD
SORBI_3001G236900 DTE..VAVKKVSHG.SRQG.........MKEFVAEVVSIGHLQHRNLVRLLGYCRRKG
SORBI_3001G237000 GTE..VAVKKVSHE.SRQG.........MKEFVAEVASIGRLRHRNLVPLLGYCRREG
SORBI_3002G350800 GLE..IAVKRVSHD.SRQG.........VREFVAEVVSIGRLRHRNLVQLLGYCRRKG
SORBI_3009G236300 KSE..VAVKRVPYN.SMQG.........TKQFVAEVVSMGHLQHNNVVTLFGYCRRKG
SORBI_3010G126900 KSE..VAVKMVPYD.SKQG.........IKQFTAEVVSLGHLQHNNIVQLHGYCRRKG
SORBI_3002G025700 KLE..VAVKKVSHE.SRQG.........IKEFIAEVVSIGRMRHRNLVPLLGYCRRRG
SORBI_3010G127300 KLE..IAVKRLSHD.SKQG.........TKEFITEIVSIGHLRHRNLVQLLGYCRRKG
SORBI_3007G038100 NVE..VAVKRMSHE.SRQG.........MKEFVTEVVSIGRLRHRNLVQLLGYCRRKG
SORBI_3002G107800 KVE..VAVKRVSHE.SRQG.........MREFIAEVVSIGRLRHRNIVPLLGYCRRQG
SORBI_3010G127200 RLN..VAVKRVSHD.SKQG.........IKEFIAEIVSIGHLQHRNLVPLLGYCRRRG
SORBI_3002G107900 KLE..VAVKRVSHD.SRQG.........MKEFIAEVVSIGRIQHRNLVRVLGYCRRRG
SORBI_3009G236200 KLE..IAVKRVSHD.SKQG.........MKEFIAEIVSIGRLRHRNLVQLLGYCRRKG
SORBI_3010G126800 KLE..VAVKRVSHE.SKQG.........MKEFIAEIVSIGRLRHRNLVQLLGYCRRKG
SORBI_3006G158266 KLE..IAVKRVSHD.SKQG.........MKEFIAEVVSIGHLRHRNLVQLLGYCRRKG
SORBI_3010G124700 GTE..VAVKRVSSSHS.STQG.........VKEFVAEVVSLGRLQHRNLVRLLGYCRRKG
SORBI_3002G024200 KLE..VAVKRVSHE.SRQG.........MKEFVAEVVSIGRIRHRNLVQLLGYCRRKG
SORBI_3007G038200 RLN..VAVKRVSHD.SKQG.........IKEFIAEIVSIGRLQNRNLVQLLGYCRREG
SORBI_3001G075000 KLE..VAVKKVSHE.SRQG.........IKEFVAEVVSIGRLRHRNLVQLLGYCRRKG
SORBI_3002G024100 KSE..IAVKKVSHN.SKQG.........MKEFVAEIVSIGRMQHPNLVRLLGYCRRKG
SORBI_3002G024300 KME..IAVKKVSHD.SKQG.........MKEFIAEVVSIGRLQHRNLVQLLGYCRRKG
SORBI_3008G139600 GET..VAVKRVNHE.SRQG.........LREFVAEIASIGRLRHRNLVQLQGWCRRRG
SORBI_3003G265300 GGD.KVAIKRISAG.TRQG.........MKEFVAEIASLGRMRHRNLVELRGWCKHGQ
SORBI_3001G310800 SGEVVVAIKRISSN.GTQG.........MREFVAEVASLGRMRHRHLVELRGWCKRGQ
SORBI_3001G315600 GDV..VAIKRISSN.GSQG.........MREFVAEVASLGRMRHRNLVELRGWCKRGQ

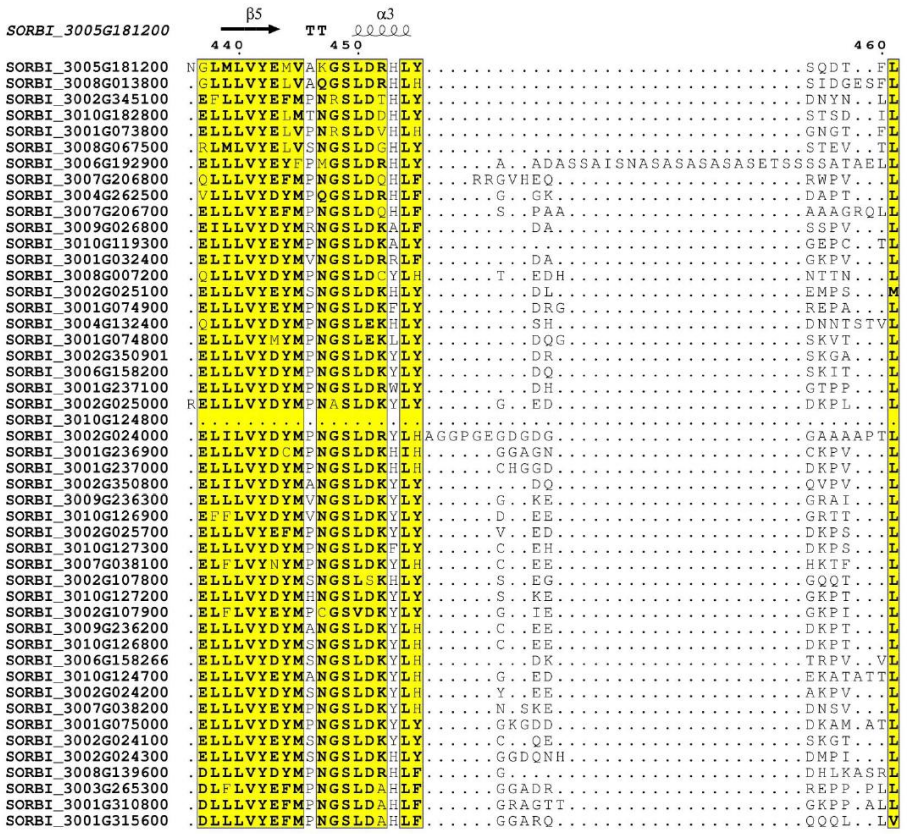
SORBI_3005G181200
β5
TT
α3
440
450
460
SORBI_3005G181200 NGLMLVYEMVAKGSLDRHLY
SORBI_3008G013800 .GLLLVYELVAQGSLDRHLH
SORBI_3002G345100 .EFLLVYEFMPNRSLDTHLY
SORBI_3010G182800 .ELLLVYELMTNGSLDDHLY
SORBI_3001G073800 .ELLLVYELVPNRSLDVHLH
SORBI_3008G067500 .RLMLVYELVSNGSLDGHLY
SORBI_3006G192900 .ELLLVYEYFPMGSLDRHLY
SORBI_3007G206800 .QLLLVYEFMPNGSLDQHLF
SORBI_3004G262500 .VLLLVYDYMPQGSLDRHLF
SORBI_3007G206700 .ELLLVYEFMPNGSLDQHLF
SORBI_3009G026800 .EILLVYDYMRNGSLDKALF
SORBI_3010G119300 .ELLLVYEYMPNGSLDKALY
SORBI_3001G032400 .ELILVYDYMVNGSLDRRLF
SORBI_3008G007200 .QLLLVYDYMPNGSLDCYLH
SORBI_3002G025100 .ELLLVYEYMSNGSLDKHLY
SORBI_3001G074900 .ELLLVYEYMPNGSLDKFLY
SORBI_3004G132400 .QLLLVYDYMPNGSLEKHLY
SORBI_3001G074800 .ELLLVYMYMPNGSLEKLLY
SORBI_3002G350901 .ELLLVYDYMPNGSLDKYLY
SORBI_3006G158200 .ELLLVYDYMPNGSLDKYLY
SORBI_3001G237100 .ELLLVYDYMPNGSLDRWLY
SORBI_3002G025000 RELLLVYDYMPNASLDKYLY
SORBI_3010G124800 .
SORBI_3002G024000 .ELILVYDYMPNGSLDRYLH
SORBI_3001G236900 .ELLLVYDCMPNGSLDKHIH
SORBI_3001G237000 .ELLLVYDYMPNGSLDKHLH
SORBI_3002G350800 .ELILVYDYMANGSLDKYLY
SORBI_3009G236300 .ELLLVYDYMVNGSLDKYLY
SORBI_3010G126900 .EFFLVYDYMVNGSLDKYLY
SORBI_3002G025700 .ELLLVYEFMPNGSLDKYLY
SORBI_3010G127300 .ELLLVYDYMPNGSLDKFLY
SORBI_3007G038100 .ELFLVYNYMPNGSLDKYLH
SORBI_3002G107800 .ELLLVYDYMSNGSLSKHLY
SORBI_3010G127200 .ELLLVYDYMHNGSLDKYLY
SORBI_3002G107900 .ELFLVYEYMPCGSVDKYLY
SORBI_3009G236200 .ELLLVYDYMANGSLDKYLH
SORBI_3010G126800 .ELLLVYDYMSNGSLDKYLH
SORBI_3006G158266 .ELLLVYDYMSNGSLDKYLH
SORBI_3010G124700 .ELLLVYEYMANGSLDKYLH
SORBI_3002G024200 .ELLLVYDYMSNGSLDKYLH
SORBI_3007G038200 .ELLLVYEYMPNGSLDKYLH
SORBI_3001G075000 .ELLLVYDYMPNGSLDKYLY
SORBI_3002G024100 .ELLLVYEYMSNGSLDKYLY
SORBI_3002G024300 .ELLLVYEYMSNGSLDKHLY
SORBI_3008G139600 .DLLLVYDYMPNGSLDRHLF
SORBI_3003G265300 .DLFLVYEFMPNGSLDAHLF
SORBI_3001G310800 .DLLLVYEFMPNGSLDAHLF
SORBI_3001G315600 .DLLLVYEFMPNGSLDAHLF

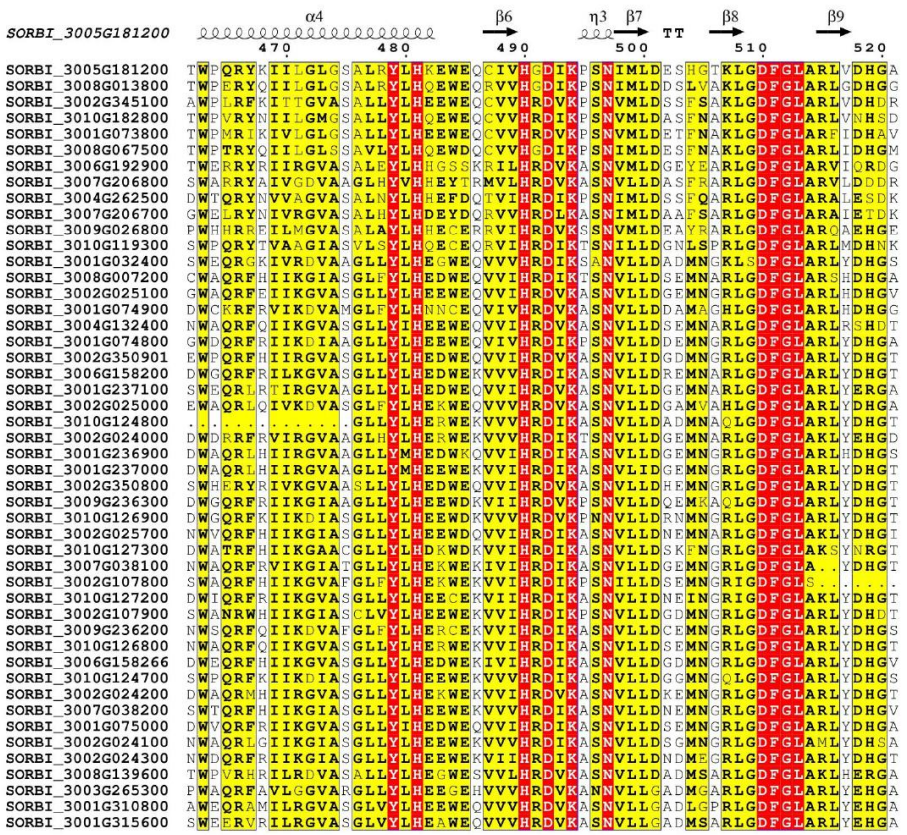
SORBI_3005G181200
α4
β6
η3
β7
TT
β8
β9
470
480
490
500
510
520
SORBI_3005G181200 TWPQRYKIILGLGSALRYLHKEWEQCIVHGDIKPSNIMLDESHGTKLGDFGLARLVDHGA
SORBI_3008G013800 TWPERYQIILGLGSALRYLHQEWEQRVVHGDIKPSNIMLDDSLVAKLGDFGLARLGDHGG
SORBI_3002G345100 AWPLRFKITTGVASALLYLHEEWEQCVVHRDVKPSNVMLDSSFSAKLGDFGLARLVDHDR
SORBI_3010G182800 TWPVRYNIILGMGSALLYLHQEWEQCVVHRDIKPSNVMLDASFNAKLGDFGLARLVNHSD
SORBI_3001G073800 TWPMRIKIVLGLGSALLYLHEEWEQCVVHRDVKPSNVMLDETFNAKLGDFGLARFIDHAV
SORBI_3008G067500 TWPTRYQIILGLSSAVLYLHQEWDQCVVHGDIKPSNIMLDESFNAKLGDFGLARLIDHGM
SORBI_3006G192900 TWERRYRIIRGVASALEYLHHGSSKRILHRDVKASNVMLDGEYEARLGDFGLARVIQRDG
SORBI_3007G206800 SWARRYAIVGDVAAGLHYVHHEYTRMVLHRDVKASNVLLDASFRARLGDFGLARVLDDDR
SORBI_3004G262500 DWTQRYNVVAGVASALNYLHHEFDQTVIHRDIKPSNIMLDSSFQARLGDFGLARALESDK
SORBI_3007G206700 GWELRYNIVRGVASALHYLHDEYDQRVVHRDLKASNIMLDAAFSARLGDFGLARAIETDK
SORBI_3009G026800 PWHHRRFILMGVASALAYLHHECERRVIHRDVKSSNVMLDFAYRARLGDFGLARQAEHGE
SORBI_3010G119300 SWPQRYTVAAGIASVLSYLHQECEQRVIHRDIKTSNILLDGNLSPRLGDFGLARLMDHNK
SORBI_3001G032400 SWEQRGKIVRDVAAGLLYLHEGWEQVVVHRDIKSANVLLDADMNGKLSDFGLARLYDHGS
SORBI_3008G007200 CWAQRFHIIKGIASGLFYLHEDWEQVVIHRDIKTSNVLLDSEMNARLGDFGLARSHDHGA
SORBI_3002G025100 GWAQRFFIIKGVASGLLYLHEEWEQVVIHRDVKASNVLLDGEMNGRLGDFGLARLHDHGV
SORBI_3001G074900 DWCKRFRVIKDVAMGLFYLHNNCEQVIVHRDVKASNVLLDDAMAGHLGDFGLARLHDHGG
SORBI_3004G132400 NWAQRFQIIKGVASGLLYIHEEWEQVVIHRDVKASNVLLDSEMNARLGDFGLARLRSHDT
SORBI_3001G074800 GWDQRFRIIKDIAAGLLYLHEEWEQVIVHRDIKPSNVLLDDEMNGRLGDFGLARLYDHGA
SORBI_3002G350901 EWPQRFHIIRGVASGLLYLHEDWEQVVIHRDVKASNVLIDGDMNGRLGDFGLARLYDHGT
SORBI_3006G158200 DWGQRFRILKGVASGLLYLHEDWEKVVVHRDIKASNVLLDREMNARLGDFGLARLYDHGT
SORBI_3001G237100 SWEQRLRTIRGVAAGLLYLHEDWEQVVIHRDVKASNVLLDGEMNARLGDFGLARLYERGA
SORBI_3002G025000 EWAQRLQIVKDVASGLFYLHEKWEQVVVHRDVKASNVLLDGAMVAHLGDFGLARLYDHGA
SORBI_3010G124800 .GLLYLHERWEKVVVHRDIKASNVLLDADMNAQLGDFGLARLYDHGT
SORBI_3002G024000 DWDRRFRVIRGVAAGLHYLHERWEKVVVHRDIKTSNVLLDGEMNARLGDFGLAKLYEHGD
SORBI_3001G236900 DWAQRLHIIRGVAAGLLYMHEDWKQVVIHRDIKASNVLLDGEMNGRLGDFGLARLHDHGS
SORBI_3001G237000 DWAQRLHIIRGVAAGLLYMHEEWEKVVIHRDIKASNVLIDGEMNGRLGDFGLARLYDHGT
SORBI_3002G350800 SWHFRYRIVKGVAASLLYLHEDWEQVVIHRDVKASNVLLDHEMNGRLGDFGLARLYDHGT
SORBI_3009G236300 DWGQRFRIIKGIAAGLLYLHEEWDKVIIHRDVKPSNVLLDQEMKAQLGDFGLARLYDHGT
SORBI_3010G126900 DWGQRFKIIKDIASGLLYLHEEWDKVVVHRDVKPNNVLLDRNMNGRLGDFGLARLYDHGT
SORBI_3002G025700 NWVQRFHIIKGIASGLLYLHEEWDQVVIHRDIKASNVLLDNEMNARLGDFGLAKLYDHGI
SORBI_3010G127300 DWATRFHIIKGAACGLLYLHDKWDKVVIHRDIKASNVLLDSKFNGRLGDFGLAKSYNRGT
SORBI_3007G038100 NWAQRFRVIKGIATGLLYLHEKWEKIVIHRDIKASNVLLDGEMNGRLGDFGLA..YDHGT
SORBI_3002G107800 SWAQRFHIIKGVAFGLFYLHEKWEKVVIHRDIKPSNILLDSEMNGRIGDFGLS.....
SORBI_3010G127200 DWIQRFRIIKGVASGLLYLHEECEKVIIHRDIKASNVLIDNEINGRIGDFGLAKLYDHGT
SORBI_3002G107900 SWANRWHIIKGIASCLVYLHEEWEKVVIHRDIKPSNVLLDGDMNGRLGDFGLARLYDHDT
SORBI_3009G236200 NWSQRFQIIKDVAFGLFYLHERCEKVVIHRDIKASNVLLDCEMNGRLGDFGLARLYDHGS
SORBI_3010G126800 NWAQRFQIIKGVASGLLYLHERWEKVVIHRDIKASNVLLDSEMNGRLGDFGLARLYDHGT
SORBI_3006G158266 DWEQRFHIIKGVASGLLYLHEDWEKIVIHRDIKASNVLLDGDMNGRLGDFGLARLYDHGV
SORBI_3010G124700 SWPQRFKIIKDIASGLLYLHEEWEKVVVHRDIKASNVLLDGGMNGQLGDFGLARLYDHGS
SORBI_3002G024200 DWAQRMHIIRGVASGLLYLHEKWEKVVIHRDVKASNVLLDKEMNGRLGDFGLARLYDHGT
SORBI_3007G038200 SWTQRFHIIKGIASGLLYLHEEWEKVVVHRDIKASNVLLDNEMNGRLGDFGLARLYDHGV
SORBI_3001G075000 DWVQRFRIIKGVASGLLYIHEDWEQVVIHRDIKASNVLLDSEMNGRLGDFGLARLYDHGA
SORBI_3002G024100 NWAQRLGIIKGIASGLLYLHEEWEKVVVHRDIKASNVLLDSGMNGRLGDFGLAMLYDHSA
SORBI_3002G024300 NWDQRFRIIKGIASGLLYLHEEWEKVIIHRDIKASNVLLDNDMEGRLGDFGLARLYDHGT
SORBI_3008G139600 TWPVRHRILRDVASALLYLHEGWESVVLHRDVKASNVLLDADMSARLGDFGLAKLHERGA
SORBI_3003G265300 PWAQRFAVLGGVARGLLYLHEEGEHVVVHRDVKANNVLLGADMGARLGDFGLARLYEHGA
SORBI_3001G310800 AWEQRAMILRGVASGLVYLHEEWEQVVVHRDVKASNVLLGADLGPRLGDFGLARLYEHGA
SORBI_3001G315600 SWEERVRILRGVASGLVYLHEAWEQVVVHRDVKASNVLLGADMSARLGDFGLARLYEHGA

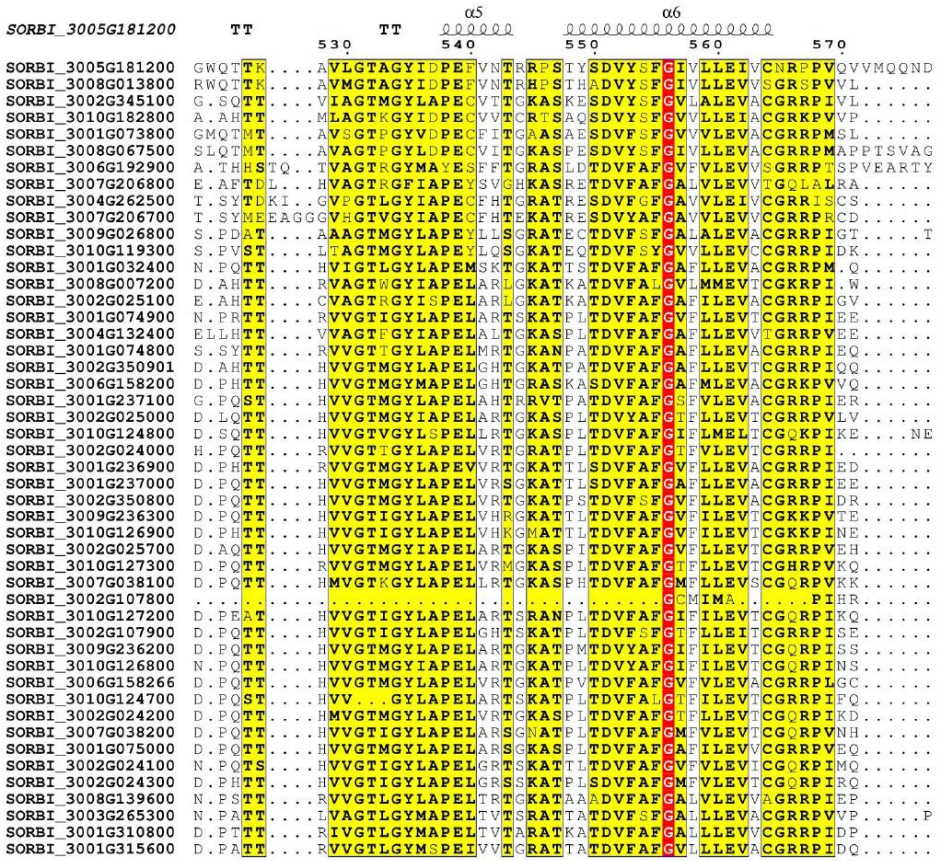
α5
α6
SORBI_3005G181200
530
540
550
560
570
SORBI_3005G181200 GWQTTK....AVLGTAGYIDPEFVNTRRPSTYSDVYSFGIVLLEIVCNRPPVQVVMQQND
SORBI_3008G013800 RWQTTK....AVMGTAGYIDPEFVNTRHPSTHADVYSFGIVLLEVVSGRSPVVL.....
SORBI_3002G345100 G.SQTT....VIAGTMGYLAPECVTTGKASKESDVYSFGVLALEVACGRRPIVL.....
SORBI_3010G182800 A.AHTT....MLAGTKGYIDPECVVTCRTSAQSDVYSFGVVLLEIACGRKPVVP.....
SORBI_3001G073800 GMQTMT....AVSGTPGYVDPECFITGAASAESDVFSFGVVVLEVACGRRPMSL.....
SORBI_3008G067500 SLQTMT....AVAGTPGYLDPECVITGKASPESDVYSFGIVLLEVACGRRPMAPPTSVAG
SORBI_3006G192900 A.THHSTQ..TVAGTRGYMAYESFFTGRASLDTDVFAFGVFVLEVVSGRRPTSPVEARTY
SORBI_3007G206800 E.AFTDL...HVAGTRGFIAPEYSVGHKASRETDVFAFGALVLEVVTGQLALRA.....
SORBI_3004G262500 T.SYTDKI..GVPGTLGYIAPECFHTGRATRESDVFGFGAVVLEIVCGRRISCS.....
SORBI_3007G206700 T.SYMEEAGGGVHGTVGYIAPECFHTERATRESDVYAFGAVVLEVVCGRRPRCD.....
SORBI_3009G026800 S.PDAT....AAAGTMGYLAPEYLLSGRATECTDVFSFGALALEVACGRRPIGT.....T
SORBI_3010G119300 S.PVST....LTAGTMGYLAPEYLQSGKATEQTDVFSYGVVLLEVCCGRRPIDK.....
SORBI_3001G032400 N.PQTT....HVIGTLGYLAPEMSKTGKATTSTDVFAFGAFLLEVACGRRPM.Q.....
SORBI_3008G007200 D.AHTT....RVAGTWGYIAPELARLGKATKATDVFALGVLMMEVTCGKRPI.W.....
SORBI_3002G025100 E.AHTT....CVAGTRGYISPELARLGKATKATDVFAFGAFILEVACGRRPIGV.....
SORBI_3001G074900 N.PRTT....RVVGTIGYLAPELARTSKATPLTDVFAFGVFLLEVTCGRRPIEE.....
SORBI_3004G132400 ELLHTT....VVAGTFGYIAPELALTGKASPLTDVFAFGAFLLEVVTGRRPVEE.....
SORBI_3001G074800 S.SYTT....RVVGTTGYLAPELMRTGKANPATDVFAFGAFLLEVACGRRPIEQ.....
SORBI_3002G350901 D.AHTT....HVVGTMGYLAPELGHTGKATPATDVFAFGAFLLEVTCGRRPIQQ.....
SORBI_3006G158200 D.PHTT....HVVGTMGYMAPELGHTGRASKASDVFAFGAFMLEVACGRKPVVQ.....
SORBI_3001G237100 G.PQST....HVVGTMGYLAPELAHTRRVTPATDVFAFGSFVLEVACGRRPIER.....
SORBI_3002G025000 D.LQTT....HVVGTMGYIAPELARTGKASPLTDVYAFGTFLLEVTCGRRPVLV.....
SORBI_3010G124800 D.SQTT....HVVGTVGYLSPELLRTGKASPLTDVFAFGIFLMELTCGQKPIKE....NE
SORBI_3002G024000 H.PQTT....RVVGTTGYLAPELVRTGRATPLTDVFAFGTFVLEVTCGRRPI.....
SORBI_3001G236900 D.PHTT....RVVGTMGYLAPEVVRTGKATTLSDVFAFGVFLLEVACGRRPIED.....
SORBI_3001G237000 D.PQTT....HVVGTMGYLAPELVRSGKATTLSDVFAFGAFLLEVACGRRPIEE.....
SORBI_3002G350800 D.PQTT....HVVGTMGYLAPELVRTGKATPSTDVFSFGVFLLEVACGRRPIDR.....
SORBI_3009G236300 D.PQTT....HVVGTIGYLAPELVHRGKATTLTDVFAFGVFILEVTCGKKPVTE.....
SORBI_3010G126900 D.PHTT....HVVGTIGYLAPELVHKGMATTLTDVFAFGVFILEVTCGKKPINE.....
SORBI_3002G025700 D.AQTT....HVVGTMGYIAPELARTGKASPITDVFAFGVFLLEVTCGRRPVEH.....
SORBI_3010G127300 D.PQTT....RVVGTMGYLAPELVRMGKASPLTDVFAFGTFLLEVTCGHRPVKQ.....
SORBI_3007G038100 D.PQTT....HMVGTKGYLAPELLRTGKASPHTDVFAFGMFLLEVSCGQRPVKK.....
SORBI_3002G107800 ..........GCMIMA.....PIHR.....
SORBI_3010G127200 D.PEAT....HVVGTIGYLAPELARTSRANPLTDVFAFGIFILEVTCGQRPIKQ.....
SORBI_3002G107900 D.PQTT....HVVGTIGYLAPELGHTSKATPLTDVFSFGTFLLEITCGRRPISE.....
SORBI_3009G236200 D.PQTT....HVVGTMGYLAPELARTGKATPMTDVYAFGIFILEVTCGQRPISS.....
SORBI_3010G126800 N.PQTT....HVVGTMGYIAPELARTGKATPLTDVYAFGIFILEVTCGQRPINS.....
SORBI_3006G158266 D.PQTT....HVVGTMGYLAPELVRTGKATPVTDVFAFGVFVLEVACGRRPLGC.....
SORBI_3010G124700 D.PQST....HVV...GYLAPELARTSKATPLTDVFALGTFILEVTCGRRPIFQ.....
SORBI_3002G024200 D.PQTT....HMVGTMGYLAPELVRTGKASPLTDVFAFGTFLLEVTCGQRPIKD.....
SORBI_3007G038200 D.PQTT....HVVGTIGYLAPELARSGNATPLTDVFAFGMFVLEVTCGQRPVNH.....
SORBI_3001G075000 D.PQTT....HVVGTMGYLAPELARSGKASPLTDVFAFGAFILEVVCGRRPVEQ.....
SORBI_3002G024100 N.PQTS....HVVGTIGYLAPELGRTSKATTLTDVFAFGVFVLEVICGQKPIMQ.....
SORBI_3002G024300 D.PHTT....HVVGTIGYLAPELGRSSKATPLSDVFAFGMFVLEVTCGQRPIRQ.....
SORBI_3008G139600 N.PSTT....RVVGTLGYLAPELTRTGKATAAADVFAFGALVLEVVAGRRPIEP.....
SORBI_3003G265300 N.PATT....LVAGTLGYMAPELTVTSRATTATDVFSFGALLLEVACGRRPVVP.....P
SORBI_3001G310800 D.PTTT....RIVGTLGYMAPELTVTARATKATDVFAFGALLLEVVCGRRPIDP.....
SORBI_3001G315600 D.PATT....RVVGTLGYMSPEIVVTGRATTATDVFAFGVLLLEVACGRRPIDQ.....
α7
α8
η4
SORBI_3005G181200
580
590
600
610
620
SORBI_3005G181200 KEAAAGEEQKQQQPPLV..L..LKWVWSLYGQSA..TLDAADERL..RGDELDEECMERV
SORBI_3008G013800 ..LQG.....EAPFV..L..LKWVWSLYGRNA..ILDAADERLRSGADELRDECMERV
SORBI_3002G345100 ..KED.....DDKIN..L..VQWVWDLYGRNE..ILNAVDGRL...DGALDEHEAVCL
SORBI_3010G182800 QEDDD.....DDKVL..L..VHWM.....
SORBI_3001G073800 MLDGQ.....KQNAVFRL..VEWVWDLYGRGA..ALAAADERL...KGEYDAAEVERV
SORBI_3008G067500 GEGKL.....QDGQVFRL..VEWAWDMHGRGG..ALDAADERL...GGAFDPWEMERV
SORBI_3006G192900 RDGGDNDEASFGRGRRQPPMHIVDWAWRLYGEGK..AQDADAAAL...CGEFDPAEVDRA
SORBI_3007G206800 .....GDTRCPLL..SDWVWQMHGRGA..LLGAVDQSL..DADEFDHDEAGRL
SORBI_3004G262500 ...NP.....AGCSQ..L..LEWVWKLHGAGR..VLEAVDPRLAAAGGGYDDEEAERL
SORBI_3007G206700 ..IEG.....FHF....L..VDWVWRLHRDGR..ALEAVDARL...DGAFDVGQAERL
SORBI_3009G026800 TDGGG.....GGRSCNNL..VEWVWSLHGEAR..LLDAMDPRL...GGEFDEDEARRA
SORBI_3010G119300 .DEGG.....GKNVN..L..VDWVWRLHGEDR..LIEAADARL...AGEFDKAEMLRL
SORBI_3001G032400 .RNDD.....VDSPG..L..VDLVLECWKAGR..IVEARDPKI....GKCDEADVELV
SORBI_3008G007200 .VNNH.....GEPLA..L..GDWVLEAWKSGL..ITHAVDPRL....DDYVEAEIELV
SORBI_3002G025100 ..NSH.....GEPQL..L..VDFVLKFWQRDL..IVCMMDPRL...EGEYVREEAELV
SORBI_3001G074900 DARGD.....GGRVL..L..VDWVLGRCSEGR..ISDCVDARL...QGEYDAGEASLA
SORBI_3004G132400 ..TID.....GDRLL..L..VDWVFEQWRKDQSLIEVVVDPRI...QGDYDVNELSLT
SORBI_3001G074800 ..GME.....GEDFA..L..VDHVLGHCLSGS..LMEAVDSKL...QDDYDAEEVCLA
SORBI_3002G350901 ..DEH.....NNRTV..L..VDWVTERWRRGL..IIDAADTMI...PDGFNPDEVSLV
SORBI_3006G158200 ..DAR.....DNRLV..L..VDWVLDRWRAGS..VTDTVDPRL...RGDFVEREASLV
SORBI_3001G237100 ..GDN.....DGRFL..L..VDWVLELWHMGA..LADAADTRL...CGDYPAEETELV
SORBI_3002G025000 .DTVH.....HGRKL..L..VDRVLECWHRGS..LEETVDSRL...QGNYNVDEARMV
SORBI_3010G124800 QGGGG.....GGHVM..L..VDWVLQHWRNGS..LVATVDAGL...RGEYDVGEAELV
SORBI_3002G024000 .....AGQTL..L..VDWVLQHWHGET..LVEAVDPRL...RGEFSSDEACLA
SORBI_3001G236900 ...NG.....GECTM..L..VEWVRGHWRNGS..ITGAMDARL.GSGSGYDAMEADMV
SORBI_3001G237000 .EEEHDAAAVGVGDRFV..L..VDWVLGHWRKGS..ITGAVDTRL...GLEYDATEADLV
SORBI_3002G350800 .DVDR.....DGRVV..L..VDWVIEHHRNGS..VLDVVDPRL...VGKYEAEEATLV
SORBI_3009G236300 ..DTQ.....GHQLM..L..VDWVIQNWHEDK..LLDSVDIRL...QGNYDVAEVCLA
SORBI_3010G126900 ..DTR.....GNQLM..L..ADWVIHNWHKDS..LLDTIDINL...QGQA.....
SORBI_3002G025700 .NREN.....NTVSM..L..VDWVLEKWHKGL..LTKVVDPRI...QDEFDINQTILV
SORBI_3010G127300 ..NEL.....GERIM..L..VDWVLDHWQQGS..LVETIDKRL....EDCNIDEACLV
SORBI_3007G038100 ..NAE.....GNEFF..L..VDWVLEHWNNGL..LTKTLDSRL...QGDYNIDEAHLV
SORBI_3002G107800 .....
SORBI_3010G127200 ..NTE.....GIQVM..L..VDWVLEHWHKGS..LTDAVDIKI...QGEYDIDEACLA
SORBI_3002G107900 ..SSQ.....ESQCM..L..VDWVLERWISGS..LLETVDSRL...QGNYNTAEACLA
SORBI_3009G236200 ..HAE.....DSSQI..L..IDWVVEHWHKGS..LTYTLDHRL...QGNCNADEVDLA
SORBI_3010G126800 ..HAE.....DSSQI..L..IDWVVKHWQEGS..LTYTMDTRL...QGSYNADEVCLA
SORBI_3006G158266 ..IAP.....DEQSV..L..LDWVQEHDRKGA..ALDTVDARL...CGKYDADEARLV
SORBI_3010G124700 .DANN.....NQQVM..L..VDWVLEHWRRGT..LVDTVDAKL...QGDFVVSEACLV
SORBI_3002G024200 ..DEQ.....GDQLL..L..VDWVLDHWHNGT..LLATVDTRL...KGNYNAEEVYLV
SORBI_3007G038200 .QNMQ.....DSQLM..L..VDWVLDKVRKGS..FSYDIGEAYLA
SORBI_3001G075000 ..SMT.....DSRLM..L..VDWVLEHWQKGS..LAEVIDAKL...QGCYDDEEAMLA
SORBI_3002G024100 ..DSD.....DNQIM..L..VDWVVEHWNKTS..LVDTVDAKL...QGDYNADEACMG
SORBI_3002G024300 ..SSN.....GDQLL..L..VDWVVEHWHHGS..LTDTVDARL...HGSYNIDEASLA
SORBI_3008G139600 .RAEP.....EELV...L..SEWAWERYAAGE..VEKVVDARL...AGTYDFAEAAAA
SORBI_3003G265300 GEATD.....DSDVI..L..VRWVRDCALDGD..LLRAVDPRL...EGCYDEGEAKLV
SORBI_3001G310800 .....VSGVN..L..LRWVWRDLGARGD..LVRAVDERL...NGRYDMEEAKLL
SORBI_3001G315600 .....ATGVN..L..VRWVWRELGAKGD..LVRAVDERL...EGRYDAGEVKLV

Supplementary Figure 2. the kinase domains in the 49 *SbLLRLK* genes.The yellow amino acid sequence indicates that the motif is conserved, and the red color represents 100% conservatism.
