# Supplementary material for: Identification, function validation and haplotype analysis of salt-tolerant genes of lectin receptor kinase gene family in sorghum (Sorghum bicolor L.)
Source: Front Genet. 2024 Oct 15;15:1464537. doi: 10.3389/fgene.2024.1464537 (PMC11518778; doi:10.3389/fgene.2024.1464537)
Supplement: Supplementary file 4 [file DataSheet4.PDF]

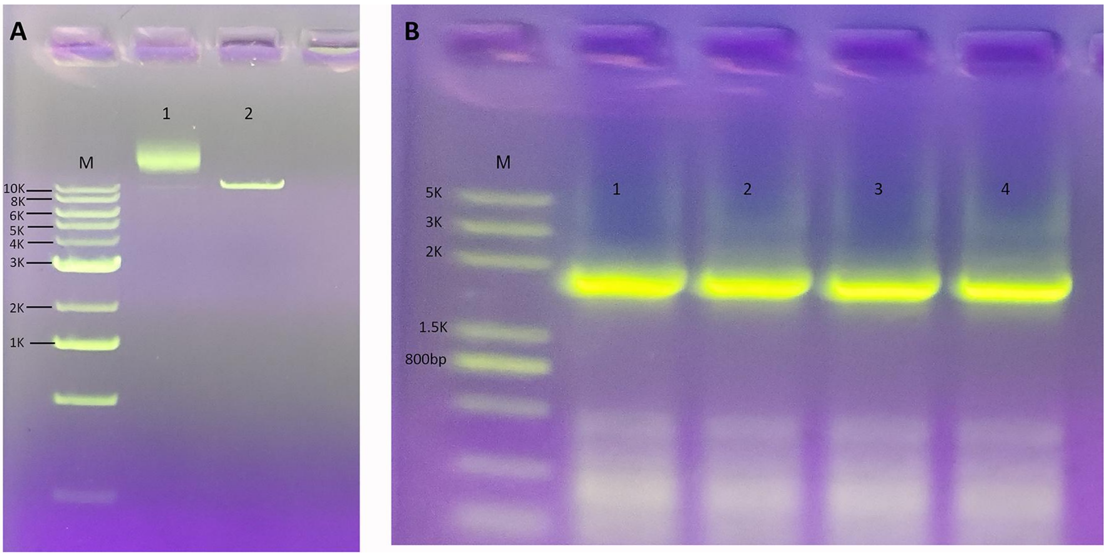


Supplementary Figure 4: Gene cloning confirmation in vector. (A) Digestion of the PCEGFP vector. Lane 1: the original vector. Lane 2: the vector digested by SpeI. (B) PCR verification of bacterial. Lane1-4: PCR verification of selected four colonies.
