# Supplementary material for: Identification, function validation and haplotype analysis of salt-tolerant genes of lectin receptor kinase gene family in sorghum (Sorghum bicolor L.)
Source: Front Genet. 2024 Oct 15;15:1464537. doi: 10.3389/fgene.2024.1464537 (PMC11518778; doi:10.3389/fgene.2024.1464537)
Supplement: Supplementary file 5 [file DataSheet6.PDF]

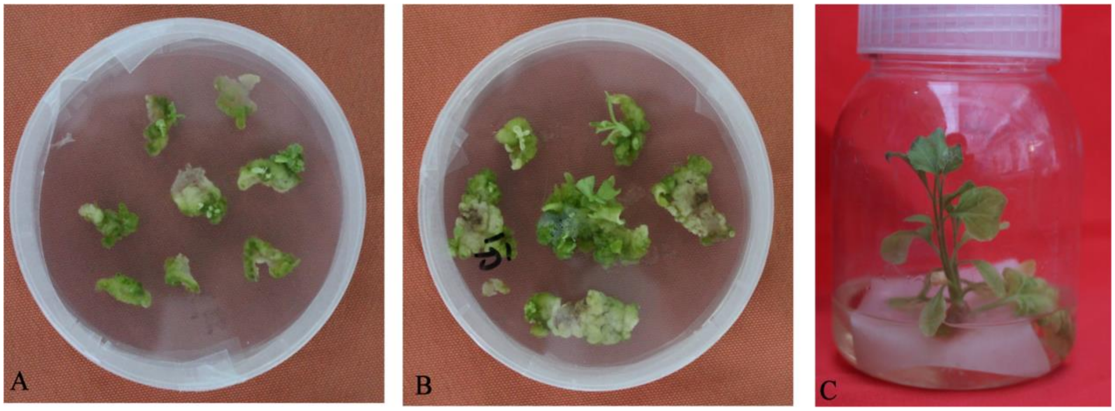

Supplementary Figure 6: Transgenic tobacco development steps. (A) Tobacco explants in the screened medium with 25mg/L hygromycin B. (B) Tobacco explants in shoot regeneration medium. (C) Tobacco regeneration shoots in rooting medium.

The transgenic development steps were listed as follows:

1. The young and healthy leaves of the tobacco seedlings were cut into 1 to 2 cm squares as the transgenic explants and placed into the Murashige and Skoog (MS) medium. The plates were incubated in a 25℃ plants growth chamber for 3 days.

2. The explants were then incubated with 2 mL of Agrobacterium GV3101 for 8 min which was transformed by the PCEGFP: SORBI_3004g304700. The explants were subsequently washed with distilled water for 3 times and placed into screened MS medium containing 25mg/L hygromycin B, 1.5mg/L 6-BA (6-Benzylaminopurine) and 200mg/L Carbethycin (Supplementary Figure 7A).

3. Four weeks later, the screened explants were transferred to shoot regeneration MS medium containing 1.5mg/L 6-BA (6-Benzylaminopurine) and 200mg/L Carbethycin (Supplementary Figure 7B).

4. Four weeks later, the regenerated plants shoots were transferred to the MS medium supplied with 0.5mg/L 1-naphthlcetic (NAA). After the roots were induced, the plants were transplanted to the soil and moved to the greenhouse at 25 ± 2°Cunder a 16 h light and 8 h dark photoperiod to generate T0 seedlings (Supplementary Figure 7C). The T2 generation of tobacco plants was used in the following experiments.
