# Supplementary material for: Identification, function validation and haplotype analysis of salt-tolerant genes of lectin receptor kinase gene family in sorghum (Sorghum bicolor L.)
Source: Front Genet. 2024 Oct 15;15:1464537. doi: 10.3389/fgene.2024.1464537 (PMC11518778; doi:10.3389/fgene.2024.1464537)
Supplement: Supplementary file 6 [file DataSheet3.PDF]

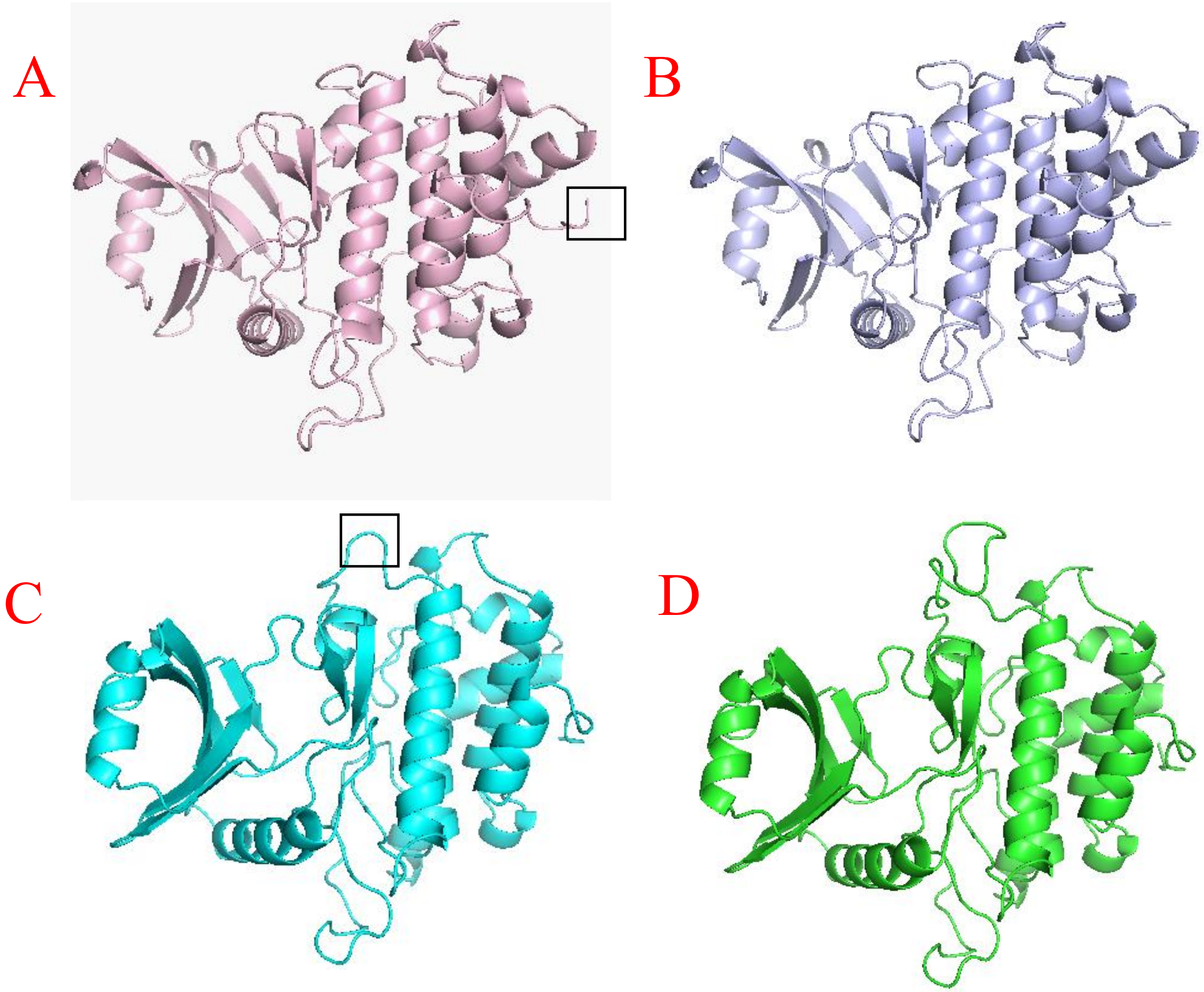

Supplementary Figure3. 3D structure of kinase domain from (A) SORBI_3004G304700; (B)SORBI_3006G158200; (C) SORBI_3001G074900 and (D) SORBI_3002G024000. 3Dstructural was similar between the duplicated gene and the original gene. The black boxesindicate structural differences.
