# Supplementary material for: Identification, function validation and haplotype analysis of salt-tolerant genes of lectin receptor kinase gene family in sorghum (Sorghum bicolor L.)
Source: Front Genet. 2024 Oct 15;15:1464537. doi: 10.3389/fgene.2024.1464537 (PMC11518778; doi:10.3389/fgene.2024.1464537)
Supplement: Supplementary file 7 [file DataSheet1.PDF]

SORBI_3005G181200

```
                                                              1          10
                                                              .           .
SORBI_3005G181200  .............................................MGTKIPV.CPSMLFF
SORBI_3008G013800  ........................................MAAAARASCGPS.GLAVLCS
SORBI_3002G345100  .................................................MRS.RTTSTCF
SORBI_3010G182800  ............................................MAMVMGSG.SSLLLSL
SORBI_3001G073800  ...................................MAGISTITSASACTAVG.IIINFFL
SORBI_3008G067500  .................................MAVKNLCSCSSSRAHHHQQ.LLLLFVF
SORBI_3006G192900  .........................................MASLSHAGIRR.RFALLAS
SORBI_3007G206800  MAIQIALGTTTVDFPLLSLSHSPFSALFLLHCAALFLPVPMAPSASCGGGRGRSPLLGCL
SORBI_3004G262500  ..............................MTTGPWGRGGRATTTTTSLALL.LLLLACV
SORBI_3007G206700  ............................................MAAARFLV.HLLLLAA
SORBI_3009G026800  ..........................................MPALASAAMA.RAAAAAA
SORBI_3010G119300  ................................................MAAA.LLRLLPF
SORBI_3001G032400  ..........................MHTRRFARRWQPPQSSRSLPRSAMPA.AALAVRV
SORBI_3008G007200  ............................................................
SORBI_3002G025100  ............................................................
SORBI_3001G074900  .................................................MPV.FVFLLCV
SORBI_3004G132400  ............................................MIEAKRVL.PLLFLYG
SORBI_3001G074800  .....................................................MPLLALA
SORBI_3002G350901  ...........................................MLFQTFHLA.TLLILIL
SORBI_3006G158200  ................................................MPPP.HLIRLLF
SORBI_3001G237100  ...........................................MRATAGLPF.LLLLFHL
SORBI_3002G025000  ..................................................MS.SFLVAFV
SORBI_3010G124800  ................................................MLFV.RLLLLIV
SORBI_3002G024000  ......................................MVVAHMPCHRRLRP.VILLSLI
SORBI_3001G236900  ................................................MAAK.TWQLMVV
SORBI_3001G237000  ..........................................MWQQVCSAMA.ANRVLPM
SORBI_3002G350800  .................................................MSA.TKLVSLV
SORBI_3009G236300  .....................................................MKLVLPI
SORBI_3010G126900  ................................................MKLI.IMTILLF
SORBI_3002G025700  ................................................MMQH.TLSLLLI
SORBI_3010G127300  ..............................................MPEKKH.KWLILCQ
SORBI_3007G038100  .................................................MKH.TSFLWYL
SORBI_3002G107800  ..............................................MHQMKH.RSFCLCF
SORBI_3010G127200  ..........................................MEKKGTDMPN.ENIMLLS
SORBI_3002G107900  ............................................MSSNTKPA.VSMLLQL
SORBI_3009G236200  ............................................................
SORBI_3010G126800  ............................................................
SORBI_3006G158266  ............................................MGRPSLPL.LPIFLLL
SORBI_3010G124700  ................................................MPQT.KITVSFL
SORBI_3002G024200  ...............................................MAHKG.VISFLTV
SORBI_3007G038200  ........................................MVMFHMKPASLM.ILKLLLV
SORBI_3001G075000  ............................................MVDFRHAR.SLLAAGV
SORBI_3002G024100  .........................................MMEHKETTMPGKKLLPISF
SORBI_3002G024300  ............................................................
SORBI_3008G139600  ............................................MSSSKKPK.PPILLSF
SORBI_3003G265300  ................................................MIAP.PNSVLCF
SORBI_3001G310800  ...............................................MSPIC.IYLFGCL
SORBI_3001G315600  ............................................MSSTSSMA.LLAHRIA
```

SORBI_3005G181200

```
                                                β1            β2
                                                ----->        -->
                          20        30        40        50
                           .         .         .         .
SORBI_3005G181200  L.............LCCSLFSTHVPRAS........SLSFNLS.FSDPASACGMQINCNN
SORBI_3008G013800  L.............YCLWTLAIHVPRAG........SVSFNLT.F....SMPQSPDLSQL
SORBI_3002G345100  L.............ALHLIVSFNITGVS........PVSFKLNFTESNNKGA......TT
SORBI_3010G182800  A.............YLLCVYVAHVTSLS.......FDYNFSIPGV...LTSA......DI
SORBI_3001G073800  S.............SVCCCYYLAAAPVA........ALSFNYDSF....GPEDLK...DI
SORBI_3008G067500  S.............LLALSLLHVPYQAN........SLSFSYTFF....NPD......DF
SORBI_3006G192900  A.............TLALLILCGTCSCL..........QFSYPSF....GTANEA...DF
SORBI_3007G206800  L..........VVVSLLCLRAMVCRAQEPGQLQPLEVATYNYTSF....QEGNSKEQREL
SORBI_3004G262500  A.............GCSISSVRAQATVFTGTVAGREITNFSFPKF....DQNRVQLATNL
SORBI_3007G206700  ASTSAVAAASTSLSTNATAAAPGVSGGN........VTGFSFSRFVSANRVV......NV
SORBI_3009G026800  T.............TSLLLLSLLHAPAA........AVRFDYASL....TLG......SL
SORBI_3010G119300  L.............LLALTAPPATAAAAVLTPAAAKNVTLDSATL....SFA......DL
SORBI_3001G032400  A.............VLLLVARGGSAAGA.......AAVEFVLTGF....AHD......NV
SORBI_3008G007200  ............................................................
SORBI_3002G025100  ......................................EFVFNGF....NDSS.....GW
SORBI_3001G074900  S.............LNLVHSSGG.............AIDFIFNGF....AGA......NI
SORBI_3004G132400  L.............HLAPAFAAGDCSSD........GDKFVYSSF....AAA......SL
SORBI_3001G074800  V.............LLASLQHAASAGAG..........QFTYNGF....AVA......SL
SORBI_3002G350901  V.............PVGRCANDATAGSD........DEQFVYNGF....KGA......NL
SORBI_3006G158200  L.............GLGGVLLPAAWAAD.........EQFVFDGF....KGA......NL
SORBI_3001G237100  H.............GLSHAAAPAAGGGG........DGQFTYNGF....TGA......GL
SORBI_3002G025000  L.............LCLGLNLPGFGDAG........NESFVFSGFPAGGADA......DL
SORBI_3010G124800  F.............LGLHPQASSDTTAD........DDQFVYSGF....RGS......AL
SORBI_3002G024000  G.............LVLLAAASAATDDD......DDGDQFVYNGF....TGA......NL
SORBI_3001G236900  V.............VMIVGMGVEPCGAV..........EFAYDGF....GGAT.....GL
SORBI_3001G237000  V.............VVAVMVAATSHGGA.........VEFAYNGF....GGA......GL
SORBI_3002G350800  V.............LLLVIHVPSSSADD........GDQFIYQGF....TAS......DL
SORBI_3009G236300  L.............LSLISEFSTVISGS.......NPPQFVYNGF....TGS......NL
SORBI_3010G126900  L.............IVGFIPEFSIADSD........HEQFVFTGF....SGS......NL
SORBI_3002G025700  P.............SLFLGINQATSSID........NAGFIYSGF....NGA......NL
SORBI_3010G127300  L.............LYLSSFKLTSFIDA........DDQFIYSSF....REA......NI
SORBI_3007G038100  L.............LLTTLASCD..............DQFLFSGF....TQS......SL
SORBI_3002G107800  I.............ISLVFAVSDFAACD........G.QFVYNGF....AGV......NL
SORBI_3010G127200  L.............LVGLSIAGFSTGED..........QFVYTGF....TSS......NL
SORBI_3002G107900  L.............FLSLNLLAAFTTGD........GQQFVYSGF....SNN......DL
SORBI_3009G236200  ............................................................
SORBI_3010G126800  ............................................................
SORBI_3006G158266  A.............AVCSDHAAMLAAAE..........EFTYNGF....GDA......NL
SORBI_3010G124700  L.............GVIHPLAALVAAGG........GDQFVYSGF....TGA......NL
SORBI_3002G024200  L.............LFHCLNPATAVTVSAAADAAANQDQFVFSGF....TGA......NL
SORBI_3007G038200  G.............IGLTIAPLSAENND........HHQFVYSGF....TNA......SL
SORBI_3001G075000  L.............LAGVVVAVLGAGED........DEHFVYSGF....TGA......PL
SORBI_3002G024100  L.............LVGLLHLVALTAGE........DDQFVYSGF....TGS......NL
SORBI_3002G024300  ............................................................
SORBI_3008G139600  L.............FLLASLAVAA............SQEFTYKGF....SASGAGKNPSL
SORBI_3003G265300  S.............VLFVLVVSSCSGDD.........VDFIYQ.................
SORBI_3001G310800  A.............VIFLSHHRLLYSVA........AVDFLYNGF....QHAA.....HL
SORBI_3001G315600  L.............FFLLARCLLRSRAD........DVDFIYNGF....RDAA.....NL
```

```
               β3      β4                                        β5          β6   β7
SORBI_3005G181200                  60          70                                 80             90
SORBI_3005G181200  TYIDGDKL.ELTRNDIVQG....TGG..........SIGRATYAKP.VPLWRAG.....
SORBI_3008G013800  ITFAGDAY..LSPNTLELTRNQRDQSSTY........SVGRATYTQP.VPLWDAA.....
SORBI_3002G345100  IQFQEDAF...YNRAIRLTKEELDGQIA.......HSVGRAVFADP.VTLWDST.....
SORBI_3010G182800  KYMSDASG...GSDRIDL....TNDTIW........STGRVAYRQP.VQLWDDA.....
SORBI_3001G073800  R.VEGDAY..ISSGWIEVTANRLSGIGH........STGRASYNAQPMRLWDKD.....
SORBI_3008G067500  R.PEDDAR...VFDGRIEL....LGDEFA......GRARGRAWHRQP.VQLWDST.....
SORBI_3006G192900  SFSPGAGI...ADGALQI....TPSTGD.....LSHRSGRVCYARETLKLWNRR.....
SORBI_3007G206800  A.FSREAR..IYKGAIQVSPDTANVGSY..QDIMVNKSGSVLLQRR.FTMWSHV..DVD
SORBI_3004G262500  T.FTGNAS..VSQGALQV....TPDSANNFERYLVNQTGRIFYSTP.FLLWASN..ASN
SORBI_3007G206700  T.VLGDAN..INQGALQITPDSLNDAAT....YLTHKSGRVLYATP.FKLWHRD.....
SORBI_3009G026800  R.LLGDAH..LKNGTIRL....SRDLPV.....PTSGAGRALYATA.VPV.........
SORBI_3010G119300  T.LLGDSF..LRNGSVGL....TRETGV.....PSSSAGTVLCTQP.VAF.RGP.....
SORBI_3001G032400  T.TSGAAV..VTSSGLLQL....TNETNE.......VFGHGFYPVP.LRF.RDD.....
SORBI_3008G007200  ..MDGEAS..VSDGLLRL....TSGQNQ........SQGHAFYTYP.LNFTSAG.....
SORBI_3002G025100  T.LQGSAV..VLPNGILNLGATNSSSVAY.......PNSLAFYPSP.FQM.RNL.....
SORBI_3001G074900  T.VDDSAM..VTPDGVLVL....TNGTYL.......VKGHGVYPAP.LHF.RSP.....
SORBI_3004G132400  T.LDGAAM..VTPRGLLQL....TSETAL.......SKGHAFHPTP.LQL.RAP.....
SORBI_3001G074800  A.VDGAAR..VAPSGLLVL....TNGTVA.......MTGHALHPSP.LQF.REP.....
SORBI_3002G350901  T.LNGDGASTVTPNGLLML....TNGTIQ.......MKGHAFHPSP.LPF.RDP.....
SORBI_3006G158200  S.FDGMAT..VTPDGLLML....TNGTNQ.......LKGHAFYPAP.LRLHRAP....N
SORBI_3001G237100  D.LDGMAV..VEPDGKLML....TNVTSQ.......LKGHAFHPAP.LRF.HDR..ASA
SORBI_3002G025000  ITLDGDGT..VTGEGLLEL....TNNETD.......SKGHAFYRNP.VQF.KDS.....
SORBI_3010G124800  T.VDGTAA..VTSDGVLLL....TNGTAY.......LKGHAFHPTP.LRL.RDS.....
SORBI_3002G024000  T.LDGSAT..VTPDGLVEL....TNGTTH.......EKGHAFHPTP.LRL.RGPPPQAG
SORBI_3001G236900  H.LDGMAT..VTPAGLLQL....TNDTSM.......PKGHAFHPEP.VTF.RRP.....
SORBI_3001G237000  S.LDGTAT..VTPAGLLQL....TNDTNM.......SKGHAFHPDP.VKFHRAP.....
SORBI_3002G350800  A.LDGLAA..VTPGGLLAL....TNATFQ.......AKAHAFRPTP.VHFLNAS.....
SORBI_3009G236300  T.VDGSAR..ITSAGLIEL....TNDTSR.......IKGHALYPSP.LRFRHSP.....
SORBI_3010G126900  T.LDGAAR..ITSTGLIEL....TNDTAR.......IKGHAFYPSP.LRFRQSP.....
SORBI_3002G025700  T.LDGMAT..ITSSGLLQL....SSGNGE.......HKGHAFHPEL.LRFHKAP.....
SORBI_3010G127300  S.LDGTAT..IKPDGILEL....TNGSFN.......LKGHAFYPTP.LHFHKSP.....
SORBI_3007G038100  N.LDGSAI..VTNGGLLDL....TNGTAI.......INGHAFYPSP.LHF.RKS.....
SORBI_3002G107800  T.LDGNAL..VTPDGLLEL....TNDTVN.......L.GHAFYPTP.LNF.SQQ.....
SORBI_3010G127200  T.LDGGAT..VTPSGLLEL....TNGTVR.......QKGHGFHPSS.LPF.YES.....
SORBI_3002G107900  L.VDGATM..ITSNGLLEL....TNGTDQ.......QIGHAFYPTP.LRFTRSP.....
SORBI_3009G236200  ...........................................................MVP...........
SORBI_3010G126800  ............................................................................
SORBI_3006G158266  S.LDGMSV..VAPNGLLVL....SNGTSQ.......MAGHAFHPAP.VRL.RDG.....
SORBI_3010G124700  T.MDGTAT..ITPGGLVEL....TNGTLQ.......LKGHAFHPTP.LSFREGG..RTG
SORBI_3002G024200  T.LDGTAT..ITADGILEL....TNGTVQ.......LKGHAFHPAP.VRL.RTP.....
SORBI_3007G038200  T.LDSTAS..ITPSGLLEL....TNGTAM.......SMGHAFYPTP.LRL.RDS.....
SORBI_3001G075000  A.LDGTAT..ITASGLLEL....TNGTAQ.......LKGHAVHPAP.LRFQRTP.....
SORBI_3002G024100  I.LDGASS..VTSSGLLEL....TNGTLR.......QKGHAIYPTQ.LRFRDQN....T
SORBI_3002G024300  ............................................................................
SORBI_3008G139600  S.LNGTSATDVLPSGVLRL....TNETSR.......LLGHAFYPAP.LRFLDRP.....
SORBI_3003G265300  ..........VLHGGALQL....TNDSNR.......LVGHAFHGSP.VRFLDVP.....
SORBI_3001G310800  S.LDGSAS..ILRGGALQL....TNDSNN.......LMGHAFFDSP.VRA.LSG.....
SORBI_3001G315600  S.LDGSAS..VLRGGALRL....TTDRGH.......VMGHAFFDSP.VRMLRGG.....
```

```
                                  β8     β9                         β10       α1
SORBI_3005G181200      TT                                    TT
                                 100         110               120         130         140
SORBI_3005G181200  .AAAGGA.....KLLASFTTSFTFRI.TP...DSSLPTGDGMAFFLTPYSSATEIPPGSG
SORBI_3008G013800  ....TGE.......TASFVTTFTFNISL....DPSTFAGDGMAFFLAHFGPGSRVPTNSS
SORBI_3002G345100  ....TGQ.......LADFTTRFTFMIKAP....VAHGSYGEGLAFFLSPYPS..VVPYNST
SORBI_3010G182800  .....GK.......VASFTSNFTFAI.KP...HNSTNQADGMAFYVGPWPP..NLPGDST
SORBI_3001G073800  ....TGE.......VASFTTRFAFVI.DPPGEHGIDNKGTGMAFFLAAAYPS.SLPSGSY
SORBI_3008G067500  ....TGE.......AASFTANFSFSIQSVPGKGGAASAGHGMAFFLAPYTP..DLPQESY
SORBI_3006G192900  ....RTA.......LTSFRTEFVLNI.VP.....RNGTGEGMAFILTNN.P..ELPRNSS
SORBI_3007G206800  GGGGGNGSATSRVQVVSFNSTFSINV.FH.LPDSSPRPGEGLTFVVAPSRD..EPPPGSY
SORBI_3004G262500  ATADGRH.......VASFSTVFQINL.YR...ANKTVKGEGLAFVVASGVD..DPPPGSD
SORBI_3007G206700  ...KANATSSGKKTVASFSTVFTVNV.FR...PNGTEPAEGFAFLIAPSTD..EPPVGSS
SORBI_3009G026800  .............RGGFSTQFAFTV.AT...LNPSSVGGGLAFVLATD....GATLGDA
SORBI_3010G119300  .....GATASNATAVASFAARFSFVIANP...NPGAAGGDGIAFFISPG....PATLGAT
SORBI_3001G032400  .ASSSSSTGAPRPQSFSFSTTFVFAM.VP...RHDDAHGHGIAFALAPS.P..TVPGAVA
SORBI_3008G007200  .VPTSSS.......VPSFSTTFVFAI.IP...QYQDLSSHGLAFVLSST.K..ELFSALP
SORBI_3002G025100  ...TDSS.......TFSFSATFAFAI.LP....YEDSARDGIAFVIAPN.T..SFTNVTR
SORBI_3001G074900  .SGAGAG.......VLSFSTTFVFAI.LS...EYAELSAYGIAFFIAPT.K..SFTDTLP
SORBI_3004G132400  ...LCGAQKKAAAAVRSFSASFVFGI.VP...VTPGMGGHGLALVVAPS.K..DLSSAMA
SORBI_3001G074800  TSNGTGT.......VRSFSASFVFAI.VG...QYLHLSSHGLAFFVSRT.R..SLSTTMP
SORBI_3002G350901  GAQNATA.......VRSFSTTFVFAI.FG...PYIDLSSHGLAFVVSSDMA..VLSTALP
SORBI_3006G158200  GSTATAA.......MQSFSTAFVIGI.IG...AYEDLSSHGMAFVVAKS.S..NFTSALP
SORBI_3001G237100  ASAQNRT.......ARSFSTTFVFAI.VS...DYVTVSGNGLAFFVAPS.K..NLSAASP
SORBI_3002G025000  ...SNGT.......VQSFSVAFVFAI.MS...AYSDFSSDGMAFVIAPG.K..DFSNASG
SORBI_3010G124800  ...PNGS.......VQSFSVAFVFGI.VS...VYPDFSAHGMALLIAPG.K..DFSSALP
SORBI_3002G024000  SPSPNGTSTTAAVPVRSFSVAFVFGI.VS...SYLDFSTHGLAFVVAPS.T..DFSTALT
SORBI_3001G236900  ...AASA.......MSSFSTTFVFAI.VS...EFLDLSTSGFAFLVAPS.T..DLSTAMP
SORBI_3001G237000  ....AGTAGAAAAAVSSFSTTFVFAI.VS...EFLDLSTSGFAFLVAPT.T..DLSTAMP
SORBI_3002G350800  .SSAAAR.......ARSFSTCFVFAI.VS...DIEGLSDQGLAFVVAPT.T..NFSAAKA
SORBI_3009G236300  ...........................................................D
SORBI_3010G126900  ....DGT.......VQSFSLSFVFGI.LS...SFGDIRGHGFAFFISPS.K..DFTDAYS
SORBI_3002G025700  ....RGK.......VQSFSVSFVFAI.LS...IAPNLSSHGMAFLISPS.T..NLSSSGT
SORBI_3010G127300  ....GGN.......VKSFSVSFIFSI.LS...AYPDKSADGMAFLVTTN.K..NFSGAFP
SORBI_3007G038100  ...PDGK.......VQSFSVNVVFSI.FI...TYPDLSADGMAFFIAPT.K..NFSDARA
SORBI_3002G107800  ...LNGS.......VQSFSVSFVFAI.LS...VHADISADGMAFFVAPT.K..NLSNTWA
SORBI_3010G127200  ....SGV.......VQSFSVSFVFAI.LS...TYPE.SGHGLAFFIAPN.K..NLSGSFP
SORBI_3002G107900  ....NGT.......VQSFSTSFVFAI.QS...VYTDLSAHGMAFIVAPS.R..NFSAALP
SORBI_3009G236200  .....................................IYPGICTDGMAFLISPT.K..DLSGAQT
SORBI_3010G126800  ......................................................MAKQA
SORBI_3006G158266  ...PGGA.......VRSFSAAFVFAI.VS...NFTVLSDNGMAFVVAPS.T..RLSTFNA
SORBI_3010G124700  TGTGIGA.......VRSFSTSFVFGI.LS...AYPDMSAHGIVFVVSPT.T..DFSTALA
SORBI_3002G024200  SSPGGGT.......VRSFSASFAFAI.QT...TYPGLSCHGIAFTVASG.T..DFSSALA
SORBI_3007G038200  ...HNST.......VQSFSAFFVFGI.IS...IYDDLSSQGLTMLIAPS....KTLSALP
SORBI_3001G075000  ....GGP.......VRSFSASFVFGI.IP...PYSDLSGHGIVFFVGKD....SFANALP
SORBI_3002G024100  TTPSGGGSSGAGGAVRSFSASFVFGI.LS...GYPDVSANGIAFFVSPT.T..DFSSAMA
SORBI_3002G024300  ............................................................
SORBI_3008G139600  ....NGT.......AVSFSTQFAFTI.AP...EFPTLGGHGFAFVVAPD.P..RMPGALP
SORBI_3003G265300  ...DGGG.....RPPSSFSTAFVLDI.VT....VGSGGGHGLAFVVAPS.T..VLPGASP
SORBI_3001G310800  ....NNA.......VVSFSTAFVFDI.VT...VGHSGGHGLAFVVAAS.K..VLPGARA
SORBI_3001G315600  .....DA.......VVSFSTAFVFQI.VT....VGRGGGAGLAFVVAAS.K..VLPGASP
```

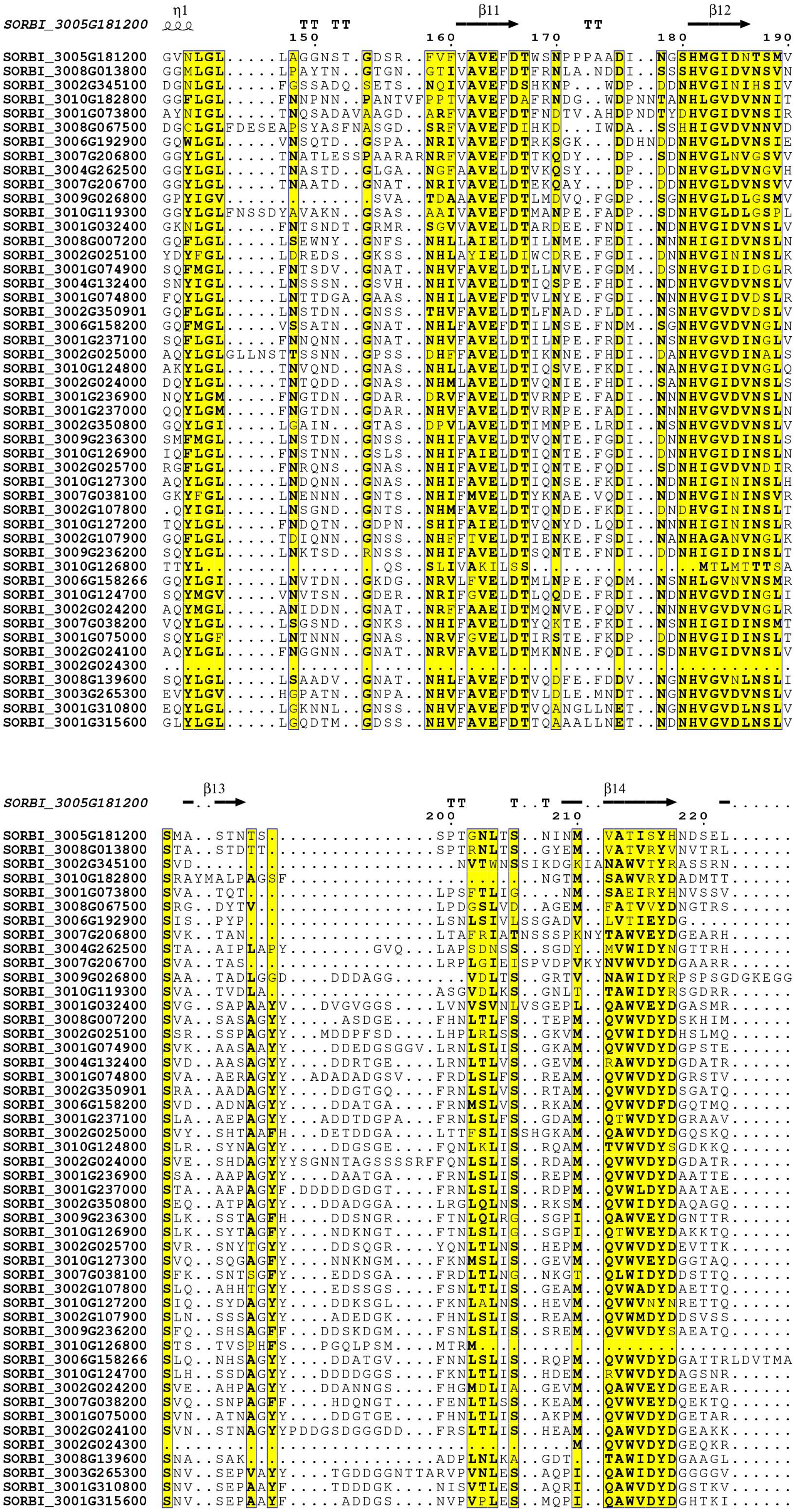

SORBI_3005G181200
η1
TT TT
β11
TT
β12
150
160
170
180
190
SORBI_3005G181200 GVNLGL.....LAGGNST.GDSR..FVFVAVEFDTWSNPPPAADI..NGSHMGIDNTSMV
SORBI_3008G013800 GGMLGL.....LPAYTN..GTGN..GTIVAVEFDTFRNLA.NDDI..SSSHVGIDVNSVN
SORBI_3002G345100 DGNLGL.....FGSSADQ.SETS..NQIVAVEFDSHKNP...WDP..DDNHVGINIHSIV
SORBI_3010G182800 GGFLGL.....FNNPNN..PANTVFPPTVAVEFDAFRNDG..WDPNNTANHLGVDVNNIT
SORBI_3001G073800 AYNIGL.....TNQSADAVAAGD..ARFVAVEFDTFNDTV.AHDPNDTYDHVGIDVNSIR
SORBI_3008G067500 DGCLGLFDESEAPSYASFNASGD..SRFVAVEFDIHKD...IWDA..SSHHIGVDVNNVD
SORBI_3006G192900 GQWLGL.....VNSQTD..GSPA..NRIVAVEFDTRKSGK...DDHNDDNHVGLDVNSIE
SORBI_3007G206800 GGYLGL.....TNATLESSPAARARNRFVAVEFDTTKQDY...DP..SDNHVGLNVGSVV
SORBI_3004G262500 GGYLGL.....TNASTD..GLGA..NGFAAVELDTVKQSY...DP..SDNHVGLDVNGVH
SORBI_3007G206700 GGYLGL.....TNAATD..GNAT..NRIVAVELDTEKQAY...DP..DDNHVGLDVNSVV
SORBI_3009G026800 GPYIGV...........SVA..TDAAAVEFDTLMDVQ.FGDP..SGNHVGLDLGSMV
SORBI_3010G119300 GGYLGLFNSSDYAVAKN..GSAS..AAIVAVEFDTMANPE.FADP..SDNHVGLDLGSPL
SORBI_3001G032400 GKNLGL.....FNTSNDT.GRMR..SGVVAVELDTARDEE.FNDI..NDNHVGIDVNSLV
SORBI_3008G007200 GQFLGL.....LSEWNY..GNFS..NHLLAIELDTILNME.FEDI..NNNHIGIDVNSLN
SORBI_3002G025100 YDYFGL.....LDREDS..GKSS..NHLAYIELDIWCDRE.FGDI..DNNHVGININSLK
SORBI_3001G074900 SQFMGL.....FNTSDV..GNAT..NHVFAVELDTLLNVE.FGDM..DSNHVGIDIDGLR
SORBI_3004G132400 SNYIGL.....LNSSSN..GSVH..NHIVAVELDTIQSPE.FHDI..NDNHVGVDVNSLV
SORBI_3001G074800 FQYLGL.....LNTTDGA.GAAS..NHILAVEFDTVLNYE.FGDI..NNNHVGIDVDSLR
SORBI_3002G350901 GQFLGL.....LNSTDN..GNSS..THVFAVELDTLFNAD.FLDI..NSNHVGVDVDSLV
SORBI_3006G158200 GQFMGL.....VSSATN..GNAT..NHLFAVEFDTILNSE.FNDM..SGNHVGVDVNGLN
SORBI_3001G237100 SQFLGL.....FNNQNN..GNAT..NHVFAVELDTILNPE.FRDI..NSNHVGVDINGLV
SORBI_3002G025000 AQYLGLGLLNSTTSSNN..GPSS..DHFFAVELDTIKNNE.FHDI..DANHVGVDINALS
SORBI_3010G124800 AKYLGL.....TNVQND..GNAS..NHLLAVELDTIQSVE.FKDI..NANHVGIDVNGLQ
SORBI_3002G024000 DQYLGL.....TNTQDD..GNAS..NHMLAVELDTVQNIE.FHDI..SANHVGIDVNSLS
SORBI_3001G236900 NQYLGM.....FNGTDN..GDAR..DRVFAVELDTVRNPE.FADI..NNNHVGVDVNSLN
SORBI_3001G237000 QQYLGM.....FNGTDN..GDAR..NHVFAVELDTVRNPE.FADI..NNNHVGVDVNSLN
SORBI_3002G350800 GQYLGI.....LGAIN...GTAS..DPVLAVELDTIMNPE.LRDI..NSNHVGVDVNSLV
SORBI_3009G236300 SMFMGL.....LNSTDN..GNSS..NHIFAVELDTVQNTE.FGDI..DNNHVGVDINSLS
SORBI_3010G126900 IQFLGL.....FNSTNN..GSLS..NHIFAIELDTIQNTE.FGDI..DNNHVGIDINSLN
SORBI_3002G025700 RGFLGL.....FNRQNS..GNAS..NHIFAVELDTIQNTE.FQDI..SDNHIGVDVNDIR
SORBI_3010G127300 AQYLGL.....LNDQNN..GNAS..NHIFAVELDTIQNSE.FKDI..NDNHIGININSLH
SORBI_3007G038100 GKYFGL.....LNENNN..GNTS..NHIFMVELDTYKNAE.VQDI..NDNHVGININSVR
SORBI_3002G107800 .QYIGL.....LNSGND..GNTS..NHMFAVELDTTQNDE.FKDI..DNDHVGININSLT
SORBI_3010G127200 TQYLGL.....FNDQTN..GDPN..SHIFAIELDTVQNYD.LQDI..NNNHIGININSLR
SORBI_3002G107900 GQFLGL.....TDIQNN..GNSS..NHFFTVELDTIENKE.FSDI..NANHAGANVNGLK
SORBI_3009G236200 SQYLGL.....LNKTSD..RNSS..NHIFAVELDTSQNTE.FNDI..DDNHIGIDINSLT
SORBI_3010G126800 TTYL...............QS..SLIVAKILSS..............MTLMTTTSA
SORBI_3006G158266 GQYLGI.....LNVTDN..GKDG..NRVLFVELDTMLNPE.FQDM..NSNHLGVNVNSMR
SORBI_3010G124700 SQYMGV.....VNVTSN..GDER..NRIFGVELDTLQQDE.FRDI..NDNHVGVDINGLI
SORBI_3002G024200 AQYMGL.....ANIDDN..GNAT..NRFFAAEIDTMQNVE.FQDV..NNNHVGVDVNGLR
SORBI_3007G038200 VQYLGL.....LSGSND..GNKS..NHIFAVELDTYQKTE.FKDI..NSNHIGIDINSMT
SORBI_3001G075000 SQYLGF.....LNTNNN..GNAS..NRVFGVELDTIRSTE.FKDP..DDNHVGIDINSLT
SORBI_3002G024100 AQYLGL.....FNGGNN..GNAT..NRVFAVELDTMKNNE.FQDI..SDNHVGIDVNSLV
SORBI_3002G024300 ....
SORBI_3008G139600 SQYLGL.....LSAADV..GNAT..NHLFAVEFDTVQDFE.FDDV..NGNHVGVNLNSLI
SORBI_3003G265300 EVYLGV.....HGPATN..GNPA..NHVFAVEFDTVLDLE.MNDT..NGNHVGVDVNSLV
SORBI_3001G310800 EQYLGL.....LGKNNL..GNSS..NHVFAVEFDTVQANGLLNET..NGNHVGVDLNSLV
SORBI_3001G315600 GLYLGL.....LGQDTM..GDSS..NHVFAVEFDTTQAAALLNET..NDNHVGVDLNSLV
SORBI_3005G181200
β13
TT
T..T
β14
200
210
220
SORBI_3005G181200 SMA..STNTS.................SPTGNLTS..NINM..VATISYHNDSEL......
SORBI_3008G013800 STA..STDTT.................SPTRNLTS..GYEM..VATVRYVNVTRL......
SORBI_3002G345100 SVD.......................NVTWNSSIKDGKIANAWVTYRASSRN......
SORBI_3010G182800 SRAYMALPAGSF......................NGTM..SAWVRYDADMTT......
SORBI_3001G073800 SVA..TQT..................LPSFTLIG....NM..SAEIRYHNVSSV......
SORBI_3008G067500 SRG..DYTV.................LPDGSLVD..AGEM..FATVVYDNGTRS......
SORBI_3006G192900 SIS..PYP..................LSNLSIVLSSGADV..LVTIEYDG..........
SORBI_3007G206800 SVK..TAN..................LTAFRIATNSSSPKNYTAWVEYDGEARH......
SORBI_3004G262500 STA..AIPLAPY.......GVQ...LAPSDNSS..SGDY..MVWIDYNGTTRH......
SORBI_3007G206700 SVA..TAS..................LRPLGIEISPVDPVKYNVWVDYDGAARR......
SORBI_3009G026800 SAA..TADLGGD....DDDAGG.......VDLTS..GRTV..NAWIDYRPSPSGDGKEGG
SORBI_3010G119300 SVA..TVDLA...............ASGVDLKS..GNLT..TAWIDYRSGDRR......
SORBI_3001G032400 SVG..SAPAAYV...DVGVGGS....LVNVSVNLVSGEPL..QAWVEYDGASMR......
SORBI_3008G007200 SVA..SASAGY....ASDGE....FHNLTLFS..TEPM..QVWVDYDSKHIM......
SORBI_3002G025100 SSR..SSPAGYY...MDDPFSD....LHPLRLSS..GKVM..QVWIDYDHSLMQ......
SORBI_3001G074900 SVK..AASAAYY...DDEDGSGGV.LRNLSLIS..GKAM..QVWVDYDGPSTE......
SORBI_3004G132400 SVD..AASAGYY...DDRTGE....LRNLTLVS..GEVM..RAWVDYDGDATR......
SORBI_3001G074800 SVA..AERAGYY..ADADADGSV..FRDLSLFS..REAM..QVWVDYDGRSTV......
SORBI_3002G350901 SRA..AADAGYY....DDGTGQ....FRNLSLVS..RTAM..QVWVDYDSGATQ......
SORBI_3006G158200 SVD..ADNAGYY...DDATGA....FRNMSLVS..RKAM..QVWVDFDGQTMQ......
SORBI_3001G237100 SLA..AEPAGYY...ADDTDGPA...FRNLSLFS..GDAM..QTWVDYDGRAAV......
SORBI_3002G025000 SVY..SHTAAFH...DETDDGA....LTTFSLISHGKAM..QAWVDYDGQSKQ......
SORBI_3010G124800 SLR..SYNAGYY....DDGSGE....FQNLKLIS..RQAM..TVWVDYSGDKKQ......
SORBI_3002G024000 SVE..SHDAGYYYSGNNTAGSSSSRFFQNLSLIS..RDAM..QVWVDYDGDATR......
SORBI_3001G236900 SSA..AAPAGYY....DAATGA....FRNLSLIS..REPM..QVWVDYDAATTE......
SORBI_3001G237000 STA..AAPAGYF.DDDDDGDGT....FRNLSLIS..RDPM..QVWLDYDAATAE......
SORBI_3002G350800 SEQ..ATPAGYY...DDADGGA....LRGLQLNS..RKSM..QVWIDYDAQAGQ......
SORBI_3009G236300 SLK..SSTAGFH...DDSNGR....FTNLQLRG..SGPI..QAWVEYDGNTTR......
SORBI_3010G126900 SLK..SYTAGFY...NDKNGT....FTNLSLIG..SGPI..QTWVEYDAKKTQ......
SORBI_3002G025700 SVR..SNYTGYY....DDSQGR....YQNLTLNS..HEPM..QVWVDYDEVTTK......
SORBI_3010G127300 SVQ..SQGAGFY...NNKNGM....FKNMSLIS..GEVM..QVWVEYDGGTAQ......
SORBI_3007G038100 SFK..SNTSGFY...EDDSGA....FRDLTLNG..NKGT..QLWIDYDDSTTQ......
SORBI_3002G107800 SLQ..AHHTGYY....EDSSGS....FSNLTLIS..GEAM..QVWADYDAETTQ......
SORBI_3010G127200 SIQ..SYDAGYY....DDKSGL....FKNLALNS..HEVM..QVWVNYNRETTQ......
SORBI_3002G107900 SLN..SSSAGYY...ADEDGK....FHNLSLIS..REAM..QVWMDYDDSVSS......
SORBI_3009G236200 SFQ..SHSAGFF...DDSKDGM....FSNLSLIS..SREM..QVWVDYSAEATQ......
SORBI_3010G126800 STS..TVSPHFS...PGQLPSM....MTRM..........................
SORBI_3006G158266 SLQ..NHSAGYY...DDDATGV....FNNLSLIS..RQPM..QVWVDYDGATTRLDVTMA
SORBI_3010G124700 SLH..SSDAGYY...DDDGGS....FKNLTLIS..HDEM..RVWVDYDAGSNR......
SORBI_3002G024200 SVE..AHPAGYY...DDANNGS....FHGMDLIA..GEVM..QAWVEYDGEEAR......
SORBI_3007G038200 SVQ..SNPAGFF....HDQNGT....FENLTLSS..KEAM..QVWVEYDQEKTQ......
SORBI_3001G075000 SVN..ATNAGYY...DDGTGE....FHNLTLIS..AKPM..QVWVDYDGETAR......
SORBI_3002G024100 SVN..STNAGYYPDDGSDGGGDD...FRSLTLIS..HEAM..QAWVDYDGEAKK......
SORBI_3002G024300 ....M..QVWVDYDGEQKR......
SORBI_3008G139600 SNA..SAK..................ADPLNLKA..GDT...TAWIDYDGAAGL......
SORBI_3003G265300 SNV..SEPVAYY....TGDDGGNTTARVPVNLES..AQPI..QAWIDYDGGGGV......
SORBI_3001G310800 SNV..SEPAAYF....TDDDGK....NISVTLES..AQRI..QAWVDYDGSTKV......
SORBI_3001G315600 SNV..SEPAAYF....ADDGS....NVPVPLES..MQPI..QAWVDYDGHTKI......

```
                  β15                β16       α2          β17                    β18
SORBI_3005G181200                                                              
                               230         240         250         260         270
SORBI_3005G181200 .LTADLLI.....NDSSYHVNTIIDLSTY.LPED.VAVGFSASTGKAGE.MHTVFNWSF
SORBI_3008G013800 .LAVQLTIN.....DDTSYYVNATVDLKSY.LPER.VAVGFSAATGAGGE.QHKVLSWTF
SORBI_3002G345100 .LSVFLTYK.DNPLFSGSSSLSYSVDLRKY.LPEK.VAIGFSAATGQLVE.AHQILYWEF
SORBI_3010G182800 .LSATLRFD.DLP.ELGLYNVSAIVDFKDAGLPPD.AAVGFSGATGDFIE.RHQILSWSF
SORBI_3001G073800 .LEMTLWLGDGRD.TPPSYNISQKVDLKSA.LPED.VSVGFSASTSTSIE.LHQLHSWYF
SORBI_3008G067500 .LDVTLMMV.GIG.SGATYTSAATVDLRSL.LPEQ.VAVGFSAATGDEHAANHTVLSFSF
SORBI_3006G192900 .AKLSIVAV.....QTYSFMYAWAGDLSQY.LTDD.ITVGFAASTGDFTE.LNQIKSWNF
SORBI_3007G206800 .VSVYIGVR.GEP.KPASPVLDSPLDLSEH.VPEQ.AYVGFTASTGTDFE.LNCILDWTL
SORBI_3004G262500 .VWVYISP..NDT.KPATAVLNASLDLSTI.LLDKTGYFGFSASTGVDYQ.LNCVKLWNM
SORBI_3007G206700 .IAVRMAVA.GKP.KPRRAVLAAPLDLGAT.VAEW.SYFGFAASTGSKYQ.LNCVLAWNM
SORBI_3009G026800 VLEVFVSYASK...RPSKPVMSAPLDLGER.VKDA.AFVGFSASTQGSTE.AHAIEWWSF
SORBI_3010G119300 .LEVFLSYA.VAT.KPKRPVLSVAVDLSPY.LKEA.MYVGFSASTEGSTQ.QHTIKEWTF
SORBI_3001G032400 .LEVTVAPA.RKP.RPSVPLVSSIVNLSSA.VADD.TYVGFTAANGAASS.SHYVLGWSF
SORBI_3008G007200 .LNVTIAPYFLFT.KPSRPLLSIAYNLSSV.LPTTTVYAGFSSSTGTLNC.KHYILGWSF
SORBI_3002G025100 .LNVSLAPI.LEP.KPKHSLLSHTIDLSQV.LLDH.MYVGFSSAASWDCY.GCFILGCSF
SORBI_3001G074900 .INVTLAPL.RMP.KPKKPLLSHVVDLSTV.ITDK.SYVGFASSLGSMSS.RHCILGWSF
SORBI_3004G132400 .IDVTLAPV.GTE.RPKKPLVSATVDLSTVIIADV.AYVGFSSSTGRLST.LHYVLGWSF
SORBI_3001G074800 .LDVTLAPV.GVP.RPKKPLLSRAVDLAAV.VPAE.AYVGFSSSTGVMAC.SHYVLGWSF
SORBI_3002G350901 .VTVTMAPL.GLA.RPKKPLLQTTVDLSGV.VQDTPAYVGFTSATGILFS.RHFVLGWSF
SORBI_3006G158200 .VNVTMAPLEAVA.RPKKPLLSTTVNLSSV.IDDT.AYVGFSSATGILFC.RHYVLGWSF
SORBI_3001G237100 .LNVTLAPV.EAP.KPKKPLISVAVDLSAV.VNDT.AYVGLSSSTGPFHT.RHYVLGWSF
SORBI_3002G025000 .LNVTLAPM.GVT.KPSKPLLSNTTDLSPV.ITDDKAFVGFSGATGPGGS.QHCVLAWSF
SORBI_3010G124800 .INVTLAPL.LMATRPARPLLSVSYDLSTV.LTDI.VYLGFTSATGRVNS.RHCVLGWSF
SORBI_3002G024000 .INVTMAPL.GMA.KPVNPLLSHVQDLSAV.LAEP.SYVGFSSATGPGNT.RHYVLGWSL
SORBI_3001G236900 .ITVTMAPA.PAPSRPQRPLLSTKIDLSTV.ITDT.AYVGFSSASSIVLV.KHYVLGWSF
SORBI_3001G237000 .VTVAMAPA.RRP.RPRRPLISTKINLSMV.ITDT.AYVGFSSASSIVLV.KHYVLGWSF
SORBI_3002G350800 .LDVTLAPV.QVP.KPTRPLISTAVDLSTIVARGP.MYVGFSSSSGVLNT.RHYVLGWSF
SORBI_3009G236300 .IHVTIAPL.GMQ.KPVTPLLSLTYNLSTV.VTEE.AYIGFSSATGLSTGHHHCVLGWSF
SORBI_3010G126900 .IDVTVAPL.GLE.KPVTPLLSLAFNLSTI.LTEE.AYIGFSSSTGLSTG.HHCILGLSF
SORBI_3002G025700 .ISVTIAPL.KMS.KPMRPLILTTYNLSTV.LTDQ.AYVGFSSTTGSIDS.QHYVLGWSF
SORBI_3010G127300 .IDVTLAPI.KMA.KPSKPLVSALYNLSTV.LTDT.AYVGFSSSTGVINS.RYCLLGWSF
SORBI_3007G038100 .INVTLAPI.NVG.KPSRPLMSTTYDLSTALSNST.SYIGFTSGASPVNS.RQYVMGWSF
SORBI_3002G107800 .IEVKLAPA.GAT.KPVRPLLSAVYNLSVI.LKDK.SYIGFSATTGAIST.RHCVLGWSF
SORBI_3010G127200 .INVTIAPL.NVA.KPVRPLLSTTYNLSTV.ITNP.AYIGFSSSTGSVSG.QHYLLGWSF
SORBI_3002G107900 .ITVTMAPL.KVA.RPKKPLFTTTYNLTSV.VTDV.AYIGFSSATGTINV.RHYVLGWSF
SORBI_3009G236200 .INVTMAPL.KVT.KPSRPLLSATYNLSTV.LEDP.SYIGFSASTGPIYS.LYCVLGWSF
SORBI_3010G126800 .INVTLAPL.RVD.KPSKPLLSATYNLSTV.LEDP.SYIGFSASTGPINS.LYCVLGWSL
SORBI_3006G158266 PVDVTMAPV.DVP.RPRKPLISAPVNLSAVGADDT.AYVGFSAATGVIYT.RHYVLGWSF
SORBI_3010G124700 .VNVTLAPL.AVA.KPRKPLISAVYNLSSV.ITDT.AYVGFSSATGSFNS.RHYVLGWSF
SORBI_3002G024200 .VDVTIAPV.GVS.KPVRPLVTTRYNLSDV.LAEP.SYVGFSSATGPINS.RHYILGWSF
SORBI_3007G038200 .IDVTMAPL.AMV.KPRRPTVFAIQNLSDV.LTDV.AYIGFSSSTGKLHT.QHYVLSWSF
SORBI_3001G075000 .INVFLTPL.GTS.KPSRPLVSATWNLSDV.LVEP.AYVGFSSATGTVKS.EHYVLGWSF
SORBI_3002G024100 .IDVTLAPL.TMG.KPARPLLSAAYDLSTV.IPDM.AYIGFSSSTGLVDS.THYVLGWSF
SORBI_3002G024300 .VDVTMAPL.RMA.KPSRPLLSTAYDLSTV.LTDV.ARVGFSSATGSFNS.RHYVLGWSF
SORBI_3008G139600 .LNVSIAN..GTAGKPAAPLISFRVDLSGV.FREQ.MYVGFSASTGVLAS.SHYVRGWSF
SORBI_3003G265300 .LNVTVAPV.SVAERPLRPLISTKLDLRPI.FREE.MYVGFSSATGKLAS.SHYILAWSF
SORBI_3001G310800 .LNVTIAPVSWQAGRPHRPLISHVIDLVPI.FKQD.MYVGFSAATGKLAS.SHYILAWSF
SORBI_3001G315600 .LNVTIAPVSVASSRPRRPLISQPVDLLPI.FKQD.MYVGFSAATGKLAS.SHYILSWSC
```

```
SORBI_3005G181200 
                                     280         290
SORBI_3005G181200 SS.TLA.....ST....SETTANVV......GTSNK......................
SORBI_3008G013800 TSTLQD.....AP....APAPAPPPALTPAGTGSSIQQPKKTS.................
SORBI_3002G345100 RSTDLQ............LKSKKMK...................................
SORBI_3010G182800 ES.TL.............TGVVVNET.QNRTTKMKNIS.......................
SORBI_3001G073800 SSSLEP.....KA....AAPILLAP.PPPAAQQRPPPPPPAPMDVSGSGRRAG.......
SORBI_3008G067500 RSTLAT.....KN....STSIAVTT.KKSTTTLQ..........................
SORBI_3006G192900 TT.LGD.....................DADDSWRHRKKVR.....................
SORBI_3007G206800 SI.........................EVI.........PEKKS.................
SORBI_3004G262500 .................TVEVLHDD.SLP...KTKTKLSGWK..................
SORBI_3007G206700 TLE........................KLPCDDDGGDGSGRRRT.................
SORBI_3009G026800 ST.ASP.....AP....SPRSAPAA.PPPESTPALPP.PVSNPVLPSPLLPGVTTPTPPA
SORBI_3010G119300 QTFGFP.....P.....TANSSFES.NATSNSSEPATVPVSNAAN...............
SORBI_3001G032400 RLGGGR.....AP....DLDLSKLP.SVPSS.RSSKKTT...S.................
SORBI_3008G007200 KLNGDA.....APLNYSALSLKAIQ.ELAQQVHARPH.SSFKA.................
SORBI_3002G025100 KV.NGK.....PS....SLDYSKLP.RVNKTKLPSPFILDYP..................
SORBI_3001G074900 CLNGSS.....AP....PLDYSKLP.MPPVAGGGGGR....SN.................
SORBI_3004G132400 AV.GGP.....AP....AIDMASLP.PLPDRRNSGSRSRPSKT.................
SORBI_3001G074800 AL.DGA.....AP....PLDVSRLP.DYP...RPSSK.PGRRR.................
SORBI_3002G350901 AF.DGP.....AP....ALNISALP.ALP...PAGPK.P..RS.................
SORBI_3006G158200 KM.NGA.....AP....ALNISALP.TLP...VTFPK.P..RS.................
SORBI_3001G237100 AV.DGA.....AP....TLDYTKLP.RMS...RVVTK.R..QS.................
SORBI_3002G025000 AV.NGP.....AP....PIDFTKIP.KLP...NSNSN.S..NSNSSHHHEAAGGV.....
SORBI_3010G124800 GK.NRP.....AP....AIDVSKLP.DLP...RAGPK.P..GS.................
SORBI_3002G024000 GTNGRP.....AP....AIDVARLP.KLPQVSSSSSSNA..RS.................
SORBI_3001G236900 SL.GGA.....AP....ALDYAKLP.KLP...RIGPK.P..RS.................
SORBI_3001G237000 AL.DGA.....AP....ALDYDKLP.KLP...RIGPK.P..RS.................
SORBI_3002G350800 SL.DGP.....AP....PLDMSKLP.VLP...RLGPK.P..RS.................
SORBI_3009G236300 GM.NSP.....AP....SIDSAKLP.KLP...YLGPR.P..PS.................
SORBI_3010G126900 GM.NSP.....AP....TIDSIKLP.KLP...YLGPR.P..PS.................
SORBI_3002G025700 AM.NQS.....AP....GIDVDKLP.KLP...HEGSK.N..SS.................
SORBI_3010G127300 SM.GNT.....TP....EIDITKLP.KLP...RVGSR.S..PS.................
SORBI_3007G038100 GM.NKP.....AP....PLDVSKLP.KLP...FHGPK.A..QS.................
SORBI_3002G107800 AM.NGP.....AP....EIDISKLP.KLP...QLGPK.P..RS.................
SORBI_3010G127200 GI.NNP.....AP....PIDITKLP.EIP...HLGQK.A..QS.................
SORBI_3002G107900 NM.SGP.....AP....SIDISRLP.ELP...RMSPK....QS.................
SORBI_3009G236200 SLGINSS....AP....QIDIAKLP.KLP...QVGPK.P..QS.................
SORBI_3010G126800 GI.NSP.....AP....LIDIAKLP.KLP...DVGPK.P..LS.................
SORBI_3006G158266 ATDGAA.....AP....ALDISKLP.ALP...RFGPK.P..RS.................
SORBI_3010G124700 AMGDVS.....AP....AIDIAKLP.KLP...RESTKAG..RS.................
SORBI_3002G024200 AM.DGP.....AP....AIDVAKLP.KLP...RLGPK.P..RS.................
SORBI_3007G038200 AM.NSP.....AP....SIDLTMLP.KLP...RLHQKGR..RS.................
SORBI_3001G075000 AM.DGP.....AP....DINVASLP.KLP...RFGPE.P..RS.................
SORBI_3002G024100 AM.DGP.....AP....AIDITKLP.KLP...REFPE.P..GS.................
SORBI_3002G024300 AM.DGP.....AP....AIDIAKLP.KLP...RFGPK.H..RA.................
SORBI_3008G139600 RLGGGA.....AP....ALDLASLP.SLP...RIKSGRN..RT.................
SORBI_3003G265300 RT.NGL.....AQ....SINLRRLP.KVP.....KPRTALSKL.................
SORBI_3001G310800 RT.GGE.....AQ....AIDLSRLP.NVP....KPPAPPPSMS.................
SORBI_3001G315600 RTGGGGGGGGLAQ....SIDLSRLP.EVP...KPPPP.PLSRS.................
```

SORBI_3005G181200

```
                                                            300         310
SORBI_3005G181200  ...............................LQLLLILLVPILSLLLCAAG.TYLFL...
SORBI_3008G013800  ...............................GGTLIIAVLVPVLFLLACAAAAVVVW...
SORBI_3002G345100  ...............................NVLVISLATSISGIICFMGL.VWCFM...
SORBI_3010G182800  ..................................LIAGLVSTGIAVLLVVAVCVCLG...
SORBI_3001G073800  ...............................VIAGASVGAALLLVLLVAVALAALLL...
SORBI_3008G067500  .................................LGAGVAAAGLLVLLLVVVAVAVLL...
SORBI_3006G192900  ...............................LLLAYLIPFAVAGTFI.....AFFVW.RR
SORBI_3007G206800  ...............................TTWVVIVAVVVPVTVVAVGV.AAFFLARK
SORBI_3004G262500  ...............................LGLVVGVPCAAALALGLLAG.LYLMM...
SORBI_3007G206700  ...............................LGLAVGVPVGVVAVVGAAVL.AYVCV...
SORBI_3009G026800  ATTGAAAAAPAVSIAPSSGFAAAGRNAGGSGTPHAAVAGAATAGAFVAASFAGVALW...
SORBI_3010G119300  ...............................SHKRVGLALGILGPVALAVAFVFFAW...
SORBI_3001G032400  ...............................QLLVVTLSLAVVVALLLASV.VVAVV...
SORBI_3008G007200  ...............................TLCIVLLPIVAISILVSAA..LAKVY...
SORBI_3002G025100  ...............................KLASRVFIFPFYIFIGSAGT.LILLVPII
SORBI_3001G074900  ...............................TVLEVVLPIGVVAFVLAAIT.SVFVF...
SORBI_3004G132400  ...............................MTLAIALPVAVGALAIIIAM.AACVL...
SORBI_3001G074800  ...............................KVLAIVLPIAAVAAAAVVTVAAVLVC...
SORBI_3002G350901  ...............................KVLNIVLPIATATLVFAVGV.LIYAV...
SORBI_3006G158200  ...............................KTLEIVLPIASAVLVFAVAA.VVFAF...
SORBI_3001G237100  ...............................KALDVVLPVAMPLLALAVLA.CVSLL...
SORBI_3002G025000  ...............................KGLEIGLPIAAFVFILTTCI.TAILL...
SORBI_3010G124800  ...............................KVLEIVLPIVTGALVLCFGG.AGVLL...
SORBI_3002G024000  ...............................KLLEIVLPLVSAAVVLAAGT.ALVLL...
SORBI_3001G236900  ...............................KALTIALPMATTVSVLAALA.VAFFL...
SORBI_3001G237000  ...............................KALTIALPIATTVSVLAAVG.VACLV...
SORBI_3002G350800  ...............................KVLDVLLPLATALLVAAVLA.AVFFV...
SORBI_3009G236300  ...............................RIMEITLPIASALFVLLTGT.IVVLL...
SORBI_3010G126900  ...............................KLLEIILPIASALLVLVIGT.TVVIL...
SORBI_3002G025700  ...............................KVMEIVMPIATAIFILVVAV.VIFQF...
SORBI_3010G127300  ...............................KVLMIVLPTAIASFILVTGT.AIILF...
SORBI_3007G038100  ...............................KLLAIVLPIATATLILSIGT.IVILV...
SORBI_3002G107800  ...............................KVLEITLPIATGLFVLAVGL.VIVLL...
SORBI_3010G127200  ...............................KALKITLPITILVLLLAASI.TIFLF...
SORBI_3002G107900  ...............................KVLQIVLPIASAAFVLAVGT.VVFLL...
SORBI_3009G236200  ...............................KVLKITLPIATAIFILIIGT.TIILF...
SORBI_3010G126800  ...............................KVLEIILPIVTATFIILLGT.TVILF...
SORBI_3006G158266  ...............................KVLEIVLPIATAAFVLALVV.VAFLW...
SORBI_3010G124700  ...............................KVLLILLPLASAAAVFCLGA.AAVLL...
SORBI_3002G024200  ...............................KVLEILLPIATALFIVTLGT.LVVML...
SORBI_3007G038200  ...............................WVLEVVLPVATAALLLSLGT.IAFLL...
SORBI_3001G075000  ...............................KVLEIVLPIATAAFVLAVVA.VVVAL...
SORBI_3002G024100  ...............................KFLEIILPIATAALVLSVGT.VLVLL...
SORBI_3002G024300  ...............................KLAEIIPPVATAAVILAVGA.IAVLL...
SORBI_3008G139600  ...............................SLILAVAFSAFVALVVLAGAGAYGAY...
SORBI_3003G265300  ...............................VIIKFAGVACAGTLTLIAAAMVIVLW...
SORBI_3001G310800  ...............................TVIKIVAVSCVATLTTIVLAIASALW...
SORBI_3001G315600  ...............................AVFKIVALSCVATLAAIVAATVAAVW...
```

SORBI_3005G181200

```
                       320       330                          340
SORBI_3005G181200  ..VRRR.....HEAASGSSDDSEE.....................QF......ELER...
SORBI_3008G013800  ..QKRR..........RRRRRSSGGIGTSDDDDN...........DHQQQDDSRAEL...
SORBI_3002G345100  ..SYKK..........TRRTRKEE.....................EE......KIEYDES
SORBI_3010G182800  ..YCRY..........LKRQSAKQIQEAPQGTDMDD.........EF......EKGT...
SORBI_3001G073800  ..QRRR..........RRREQAQELEDIFGGSDDDEPIM......EI......ELGT...
SORBI_3008G067500  ..VRRS..........RRRGKQRYDEEKLTTDGDDDDSLDVVDDRDF......ESST...
SORBI_3006G192900  LTRPRR..........LAYRNLEK.....................MI......DAQ....
SORBI_3007G206800  LRARRS..........MERRQERL.............................EQQL...
SORBI_3004G262500  ..KKRK..........KQVGDDPS.............................SVVF.HN
SORBI_3007G206700  ..VKRR..........KVHGDDGG.............................NSSSAIT
SORBI_3009G026800  .ALARRARARKRGQTAVAVATSKR.....................DSGVASAAALAR...
SORBI_3010G119300  VSIKKL..........IQLTSRNDA....................AF......SPEL...
SORBI_3001G032400  ..VVRR..........RRFAEEEE.....................DW......EIEY...
SORBI_3008G007200  ..MKRQ..........LQARKTEL.....................EW......QREY...
SORBI_3002G025100  LAVRRC..........YRYREIRE.....................DW......ELEF...
SORBI_3001G074900  ..GWRR..........VKYAELRE.....................EW......EDEF...
SORBI_3004G132400  ..LVVR..........RRYIELRE.....................DW......EIEF...
SORBI_3001G074800  ..VRRR..........MEYAELRE.....................DW......EAEF...
SORBI_3002G350901  ..VRRR..........IKFAELRE.....................DW......ETAF...
SORBI_3006G158200  ..LRRR..........RMYAEVKE.....................EW......EASF...
SORBI_3001G237100  ..AWRR..........FRYAELRE.....................EW......EVEF...
SORBI_3002G025000  ..VRRH..........LTYAELRE.....................DW......EVEF...
SORBI_3010G124800  ..VRRR..........FRYAELRE.....................DW......EVEF...
SORBI_3002G024000  ..VRRR..........LRYAEVRE.....................DW......ETEF...
SORBI_3001G236900  ..LRQR..........LRYAELRE.....................DW......EVEF...
SORBI_3001G237000  ..LRRR..........LRYAELRE.....................DW......EVEF...
SORBI_3002G350800  ..VWRR..........RRFAEVRE.....................DW......EDEF...
SORBI_3009G236300  ..VRRH..........FRYKEVHE.....................DW......EVEY...
SORBI_3010G126900  ..IRRH..........FRYKEIRE.....................DW......EVEY...
SORBI_3002G025700  ..MWRR..........LKYAELRE.....................DW......EVEF...
SORBI_3010G127300  ..ARRK..........LANDELQE.....................DW......EVEF...
SORBI_3007G038100  ..IQRR..........LKYAEVRE.....................DW......EAEF...
SORBI_3002G107800  ..VYRR..........LKYKEVKE.....................DW......EVEF...
SORBI_3010G127200  ..MRRN..........LRYAELRE.....................DW......EDEY...
SORBI_3002G107900  ..VRRH..........LKYAELRE.....................DW......EVEF...
SORBI_3009G236200  ..VRRQ..........MKFAEIKE.....................DW......EDEF...
SORBI_3010G126800  ..VRRR..........MKYAEVQE.....................DW......EAEF...
SORBI_3006G158266  ..VRRR..........VRYAEVRE.....................DW......EVEF...
SORBI_3010G124700  ..VRRR..........RKYTELRE.....................EW......EVEF...
SORBI_3002G024200  ..VRRR..........MRYAELRE.....................DW......EVDF...
SORBI_3007G038200  ..LRRH..........FRYAEVRE.....................DW......ELEF...
SORBI_3001G075000  ..VRRR..........LKYAELRE.....................DW......EVEF...
SORBI_3002G024100  ..RRRQ..........LRYTELRE.....................DW......ELEF...
SORBI_3002G024300  ..VRRR..........LRYTEVRE.....................DW......EVEF...
SORBI_3008G139600  .....R..........YKNRDIIE.....................PW......ELDY...
SORBI_3003G265300  ..LRKR..........AKLADTLE.....................DW......ELEH...
SORBI_3001G310800  ..LRRR..........AILAETLE.....................EW......ERDH...
SORBI_3001G315600  ..LRQR..........AALAETLE.....................EW......ELDH...
```

Supplementary Figure 1. the lectin domains in the 49 *SbLLRLK* genes.The yellow amino acid sequence indicates that the motif is conserved, and the red color represents 100% conservatism.
