# Supplementary material for: Identification, function validation and haplotype analysis of salt-tolerant genes of lectin receptor kinase gene family in sorghum (Sorghum bicolor L.)
Source: Front Genet. 2024 Oct 15;15:1464537. doi: 10.3389/fgene.2024.1464537 (PMC11518778; doi:10.3389/fgene.2024.1464537)
Supplement: Supplementary file 9 [file DataSheet8.docx]

Supplementary Figure 1: the lectin domains in the 49 *SbLLRLK* genes.

Supplementary Figure 2. the kinase domains in the 49 *SbLLRLK* genes.

Supplementary Figure 3. 3D structure of kinase domain from (A) SORBI_3004G304700;

(B)SORBI_3006G158200; (C) SORBI_3001G074900 and (D) SORBI_3002G024000.

Supplementary Figure 4: Gene cloning confirmation in vector.

Supplementary Figure 5: The cloning strategy in this study.

Supplementary Figure 6: Transgenic tobacco development steps.

Supplementary Figure 7: PCR and QPCR confirmation of transgenic tobacco lines.

Supplementary Table: The research data related to this study.
