# Supplementary material for: Identification, function validation and haplotype analysis of salt-tolerant genes of lectin receptor kinase gene family in sorghum (Sorghum bicolor L.)
Source: Front Genet. 2024 Oct 15;15:1464537. doi: 10.3389/fgene.2024.1464537 (PMC11518778; doi:10.3389/fgene.2024.1464537)
Supplement: Supplementary file 8 [file DataSheet5.PDF]

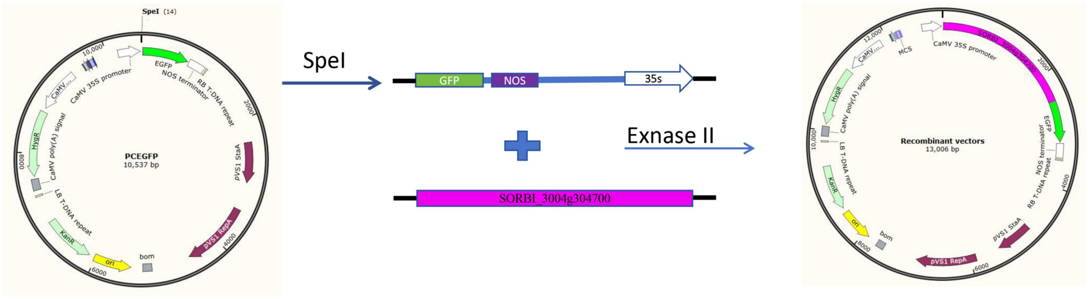

Supplementary Figure 5: The cloning strategy in this study.

Firstly, the PCEGFP vector was digested and linearized by using SpeI. The purified digested vector was mixed with the purified product of the target gene (SORBI_3004g304700). Both the 5' and 3' ends of the linearized vector and the purified product of the target gene contained 16-bp homologous sequences (depicted as black squares in the diagram). Subsequently, the mixture was subjected to homologous recombination connection catalyzed by Exnase(Vazyme,China) enzyme, resulting in the generation of the recombinant vector.
